# Evolutionary and functional analysis of mulberry type III polyketide synthases

**DOI:** 10.1186/s12864-016-2843-7

**Published:** 2016-08-04

**Authors:** Han Li, Jiubo Liang, Hu Chen, Guangyu Ding, Bi Ma, Ningjia He

**Affiliations:** State Key Laboratory of Silkworm Genome Biology, Southwest University, Beibei, Chongqing, 400715 People’s Republic of China

**Keywords:** Chalcone synthase, Stilbene synthase, Functional analysis, Evolutionary analysis, Gene expression, Mulberry

## Abstract

**Background:**

Type III polyketide synthases are important for the biosynthesis of flavonoids and various plant polyphenols. Mulberry plants have abundant polyphenols, but very little is known about the mulberry type III polyketide synthase genes. An analysis of these genes may provide new targets for genetic improvement to increase relevant secondary metabolites and enhance the plant tolerance to biotic and abiotic stresses.

**Results:**

Eighteen genes encoding type III polyketide synthases were identified, including six chalcone synthases (*CHS*), ten stilbene synthases (*STS*), and two polyketide synthases (*PKS*). Functional characterization of four genes representing most of the *MnCHS* and *MnSTS* genes by coexpression with 4-Coumaroyl-CoA ligase in *Escherichia coli* indicated that their products were able to catalyze *p*-coumaroyl-CoA and malonyl-CoA to generate naringenin and resveratrol, respectively. Microsynteny analysis within mulberry indicated that segmental and tandem duplication events contributed to the expansion of the *MnCHS* family, while tandem duplications were mainly responsible for the generation of the *MnSTS* genes. Combining the evolution and expression analysis results of the mulberry type III *PKS* genes indicated that *MnCHS* and *MnSTS* genes evolved mainly under purifying selection to maintain their original functions, but transcriptional subfunctionalization occurred during long-term species evolution. Moreover, mulberry leaves can rapidly accumulated oxyresveratrol after UV-C irradiation, suggesting that resveratrol was converted to oxyresveratrol.

**Conclusions:**

Characterizing the functions and evolution of mulberry type III *PKS* genes is crucial for advancing our understanding of these genes and providing the basis for further studies on the biosynthesis of relevant secondary metabolites in mulberry plants.

**Electronic supplementary material:**

The online version of this article (doi:10.1186/s12864-016-2843-7) contains supplementary material, which is available to authorized users.

## Background

Mulberry (*Morus* spp.), belonging to the order Rosales, family Moraceae, and genus *Morus*, has been used as a food product and in herbal medicines in China for over 1,900 years [1]. Different plant parts, such as leaves, fruits, branches, bark, roots, and shoots, have a variety of pharmacological effects, including preventing headaches, hypertension, and diabetes, in addition to act as a diuretic [2]. The pharmacological effects of mulberry are attributed to bioactive compounds in tissues. Flavonoids and stilbenes are representative bioactive compounds with a wide range of activities, including UV protection, defense against herbivores and pathogens, and protection from abiotic stresses [3–5]. Their potential medicinal properties have also resulted in their use in treatments for a variety of diseases. For example, anthocyanins, which are a group of water-soluble pigments in mulberry fruits, are beneficial to humans in treatments for obesity, inflammation, and cancer [6]. Oxyresveratrol, which is a representative mulberry stilbene, has been used in cosmetics and for the treatment of hyperpigmentation disorders [7].

There are two pathways for the biosynthesis of flavonoids and stilbenes, which begins with the condensation of one CoA-ester molecule of cinnamic acid or derivatives such as coumaric or ferulic acid, and three molecules of malonyl-CoA (Additional file 1: Figure S1) [8]. The generated tetraketide intermediate is subsequently folded and cyclized to form a chalcone or stilbene ring structure depending on the polyketide synthase activities of chalcone synthase (CHS) or stilbene synthase (STS) [9]. Chalcone synthase catalyzes a C6 → C1 Claisen condensation of the elongated tetraketide intermediate to form naringenin chalcone, while STS catalyzes an alternative C2 → C7 aldol condensation of the same tetraketide intermediate to produce a stilbene backbone [10]. Both CHS and STS belong to the plant type III polyketide synthase superfamily, and share considerable similarities in their sequences and crystallographic structures, suggesting that STS independently evolved from CHS several times [11, 12]. Unlike CHS, which is present in all higher plants, STS is restricted to a few species such as peanut (*Arachis hypogaea*), Scots pine (*Pinus sylvestris*), and grapevine (*Vitis vinifera*) [13–15]. Besides CHS and STS, a growing number of functionally divergent CHS-like type III *PKS* genes have been cloned and characterized in plants. For example, 2-pyrone synthase (2-PS) from *Gerbera hybrida* catalyzes the formation of 6-methyl-4-hydroxy-2-pyrone [16]. Acridone synthase (ACS) from *Ruta graveolens* catalyzes three condensations of malonyl-CoA with an N-methylanthraniloyl-CoA starter to form a three-ring acridone skeleton of acridone alkaloids [17]. Other members in plant type III polyketide synthase superfamily, such as benzalacetone synthase (BAS), styrylpyrone synthase (SPS), and 4-coumaroyl triacetic acid synthase (CTAS), also catalyze iterative decarboxylative condensations of malonyl unit with a CoA-linked starter molecule to produce structurally diverse, pharmaceutically important plant secondary metabolites [10]. It is clearly evident that gene families arose by gene duplication and subsequent sequence divergence [18]. In some cases, more gene family members help to increase the abundance of enzymes and/or proteins [19]. In other instances, duplicate genes can lead to new functions [20].

Although flavonoids and stilbenes have important roles in mulberry plants, little is known about their biosynthesis in these plants. In this study, we identified and analyzed type III polyketide synthases in the genome of the mulberry tree *Morus notabilis* [21]. Our analyses of gene organization and expression, molecular evolution, and functions have generated new insights into the roles of mulberry type III polyketide synthases, and have laid the foundation for future studies on secondary metabolite biosynthesis in mulberry plants.

## Results

### Identification and phylogenetic analysis of mulberry type III polyketide synthases

**Fig. 1:**
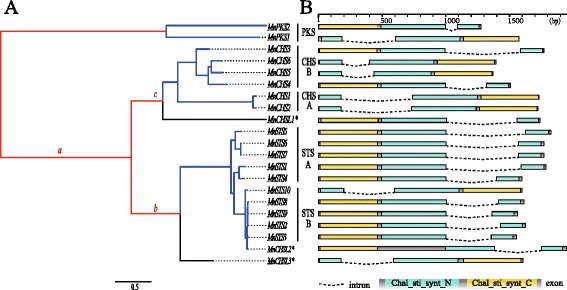
Mulberry type III polyketide synthase superfamily. **a** Phylogenetic relationships among the type III *PKS* genes. Branch length is defined as the number of nucleotide substitutions per codon according to PAML. Different colors indicate higher posteriori probabilities of evolving under different selection regimens: red and blue correspond to positive and purifying selections, respectively, while black indicates the selection constraints of the branch are relaxed relative to the other branches of the same clade. The lowercase letters refer to the following: a, branch ancestral to the *MnCHS* and *MnSTS* families; b, branch ancestral to the *MnSTS* family clade; c, branch ancestral to the *MnCHS* family clade. *: genes without expression data (**b**) Intron/exon structure of the type III *PKS* genes. Boxes: exon; black line: intron; cyan boxes: N-terminal chalcone/stilbene synthase domain (IPR001099); yellow boxes: C-terminal chalcone/stilbene synthase domain (IPR012328)

Twenty-one putative type III polyketide synthase genes (*PKS*) were identified based on the recently sequenced *M. notabilis* genome (see Methods). The genes included six *CHS* (*MnCHS1–6*) genes, ten *STS* (*MnSTS1–10*) genes, two *PKS* genes (*MnPKS1–2*), and three genes with undetectable expression (*MnCHSL1–3)* (Fig. 1, Additional file 2: Table S1). In addition, six pseudogenes that were disrupted by stop codons, frame-shifts, and/or small deletions were also identified (data are not shown). For *MnCHS* gene family, *MnCHS1* and *MnCHS2* were identified and analyzed in one of our previous studies [22]. The deduced length of the enzymes encoded by these genes was 389 amino acids. Most of the remaining four *MnCHS* genes encoded a 391-amino acid enzyme, except *MnCHS4*, whose enzyme was predicted to consist of 394 amino acids. The *MnSTS* genes encoded enzymes with 399 residues, and exhibited a highly conserved gene structure. All *MnSTS* genes contained two exons separated by an intron, following the GT/AG rule (Fig. 1).

**Fig. 2:**
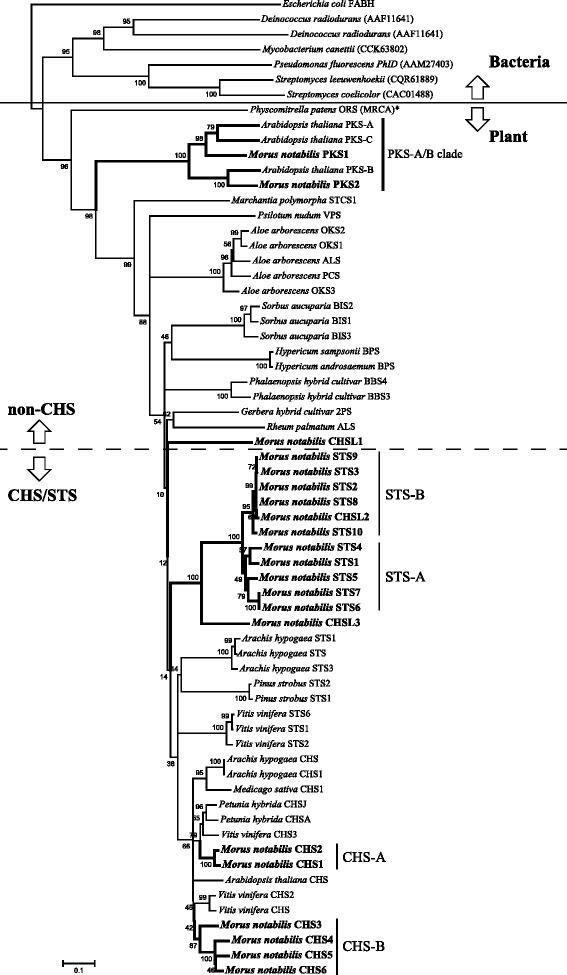
Phylogenetic analysis of mulberry type III polyketide synthases and other type III polyketide synthases. The indicated scale represents 0.1 amino acid substitutions per site. ALS, aloesone synthase; BBS, bibenzyl synthase; BPS, benzophenone synthase; STCS, stilbenecarboxylate synthase; VPS, valerophenone synthase; OKS, octaketide synthase; 2PS, 2-pyrone synthase; ORS, 2′- oxoalkylresorcinol synthase. The asterisk indicates the branch that is the most recent common ancestor to the plant CHS family. The *β*-ketoacyl carrier protein synthase III (FABH) of *Escherichia coli* was used as an outgroup

Phylogenetic analyses revealed that mulberry CHS and STS clustered together with other plant CHS or STS homologs (Fig. 2). Each mulberry CHS and STS family can be divided into two subgroups, namely MnCHS-A, MnCHS-B, MnSTS-A, and MnSTS-B. Similar phylogenetic analysis result based on nucleotide sequences of mulberry type III PKSs also supported such clustering (Fig. 1). MnCHSL2 and MnCHSL3 located in plant CHS/STS clade were further clustered with mulberry STS proteins, suggesting they shared a closer evolutional relationship. MnCHSL1, which was outside the CHS/STS clade, shared more sequences identical with MnCHSs (66.84 % ~ 68.21 %) than MnSTSs (56.52 % ~ 57.54 %). Two mulberry PKS proteins were located in a PKS-A/B clade containing three PKSs from *Arabidopsis thaliana*, which produce long-chain alkyl α-pyrones and participate in the biosynthesis of sporopollenin [23]. This PKS-A/B clade was distant to the plant CHS/STS cluster, but close to the *Physcomitrella patens* 2′-oxoalkylresorcinol synthase, which is considered the most recent common ancestor of the plant CHS family [24].

The amino acid sequences of MnPKS1 and MnPKS2 were 64.7 % identical, and were more similar to those of AtPKS-A (78.12 %) and AtPKS-B (82.35 %), respectively, than to those of other plant type III polyketide synthases (less than 40 %). MnPKS1 and MnPKS2 contained CHS active site residues, including Gly211, Phe215, Gly216, Gly256, Phe265, Ser338, and Pro375, as well as conserved catalytic residues such as Cys-164, His-303, and Asn-336 (Additional file 3: Figure S2) [8]. The Thr197 residue was substituted by Gly in MnPKS1 and MnPKS2 (Additional file 3: Figure S2), which was consistent with AtPKS-A and AtPKS-B sequences. This residue regulates product chain length by sterically modifying the active-site cavity, suggesting the function of MnPKS1 and MnPKS2 is related to that of the AtPKS-A and AtPKS-B enzymes [25–27].

### Expansion patterns of mulberry type III *PKS* gene families

**Fig. 3:**
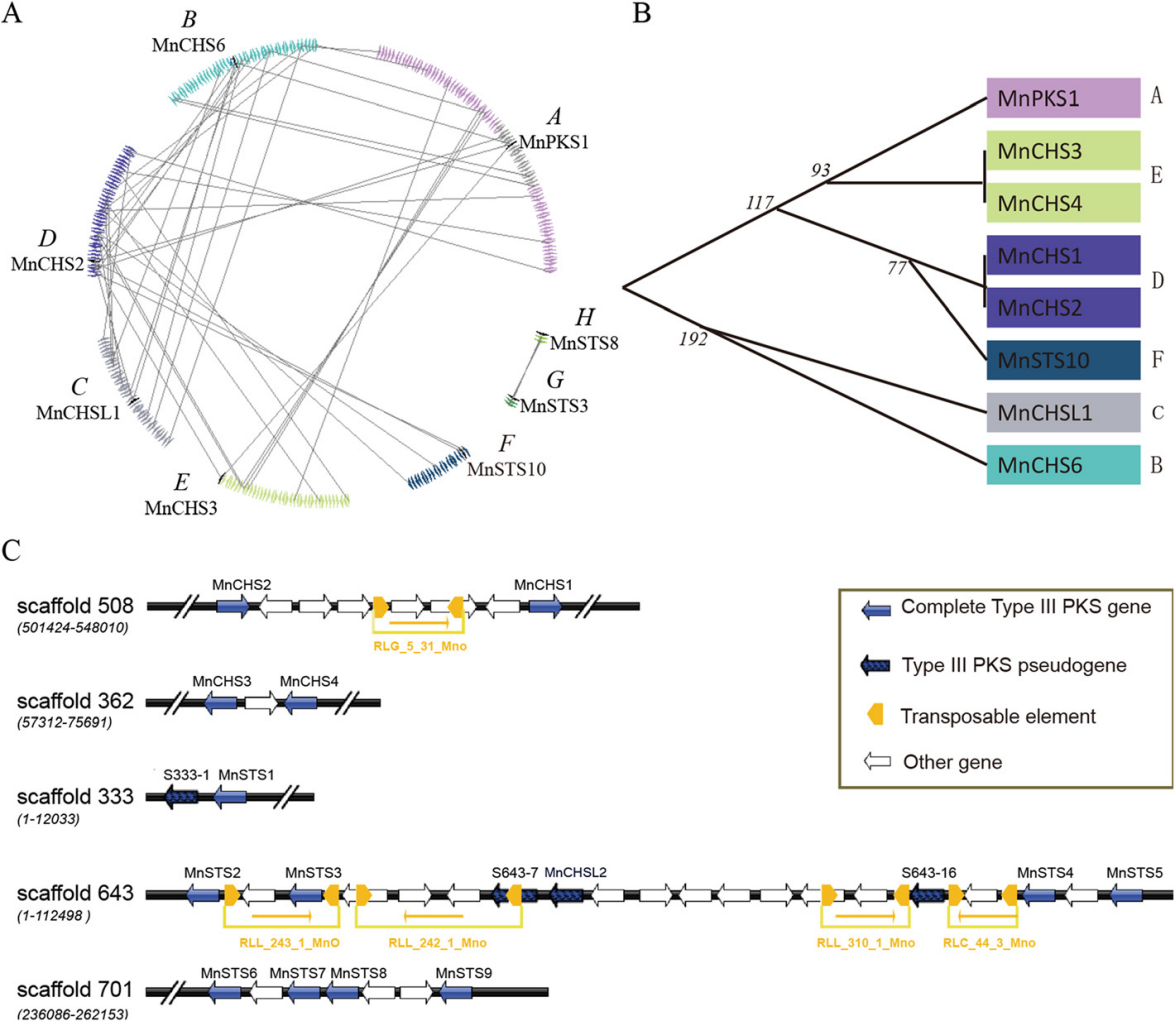
Evolutionary relationships among mulberry type III polyketide synthases. **a** Genes represented by a series of triangles of the same color are from the same genomic fragment. Genes from the type III *PKS* superfamily are indicated by black triangles. Triangles also indicate gene orientation on strands. The colinear homologous genes are linked by gray lines. **b** Evolutionary relationships were determined to clarify the order of duplication events for the type III polyketide synthase superfamily. The black vertical line indicates tandem duplication. Numbers indicate when (million years ago, MYA) the segmental duplication events occurred. **c** Organization of the mulberry type III *PKS* gene clusters. The long terminal repeat retrotransposons are presented in yellow. Right and left arrows indicate whether a retrotransposon is located on the + or − strand, respectively. RLG: retrotransposon belongs to the *Gypsy* superfamily; RLL: retrotransposon belongs to the *Lard* superfamily. Numbers represent the sequence ID

*MnCHS6* shared a substantial colinear region with *MnPKS1*, *MnCHSL1*, and *MnCHS2/MnCHS1* (Fig. 3a and c), suggesting these genes are linked by segmental duplication events. Conserved colinear genes surrounding *MnPKS1*, *MnCHS2/MnCHS1*, and *MnCHS3/MnCHS4* were also detected. Less conserved colinear genes were detected between segments D and F. Although segments H and G contained a less conserved colinear region, nine of ten *MnSTS* genes originated in tandem arrays, suggesting tandem duplications were primarily responsible for generating the *MnSTS* family. *MnSTS* genes located on scaffold-643 were mixed with pseudogenes and some relics of transposable elements. The predicted syntenic relationships and levels of synonymous substitution (Ks) in duplicated pairs (Additional file 2: Table S2) indicated that *MnPKS1* and *MnCHS6* arose from a single ancient duplication event, and *MnCHSL1* arose after this duplication event. A detectable colinear relationship among the D, E, and A segments suggests *MnCHS2/CHS1* and *MnCHS3/CHS4* evolved from *MnPKS1* after the appearance of *MnCHSL1*. Segments G and H, as outliers, lacked significant colinear relationships with the other segments (Additional file 2: Table S2). Additionally, based on the Ks values of all pairs of protein-coding genes among all genomic fragments, we used synonymous substitution rate of plant nuclear genes (5 × 10^−9^) to estimate the time when the segmental duplication events took place [28]. *MnCHSL1* and *MnCHS6* diverged about 192 million years ago (mya), which is approximate the divergence of dicots and monocots (around 200 mya) and is much earlier than the divergence times of the remaining *MnPKS* genes, including *MnPKS1* and *MnCHS2*/*MnCHS1* (117 mya), *MnCHS3*/*MnCHS4* and *MnPKS1* (93 mya), *MnCHS2* and *MnSTS10* (77 mya), and *MnSTS3* and *MnSTS8* (37 mya) (Fig. 3b, Additional file 2: Table S2) [29].

### Selection constraints on the coding sequence of mulberry type III *PKS*

To explore how selective constraints evolved following the duplication of mulberry type III *PKS* genes, we calculated the *d*_*N*_*/d*_*S*_ ratio (ω) using various codon substitution models, including the Branch, Clade, and Branch site models. Two data sets were analyzed independently. One set includes *PKS*, *CHS* and *STS* sequences from different plant species (Additional file 4: Figure S3). The other set is restricted to the type III *PKS* sequences identified in the mulberry genome (Fig. 1). As presented in Table 1, comparisons of complex Branch models with simpler models (i.e., MnCHS and M0) and nested models (i.e., MnCHS&MnSTS and MnCHS-MnSTS) indicated that the ω values for *MnCHS* and *MnSTS* sequences in mulberry sequence set (MSS) were <1: *MnCHS* ω = 0.0689 [likelihood ratio test (LRT) *P* value approximately 1.721× 10^−5^] and *MnSTS* ω = 0.1630 (LRT *P* value approximately 8.220× 10^−7^) in simpler models; *MnCHS* ω = 0.0703 and *MnSTS* ω = 0.1613 (LRT *P* value approximately 2.290× 10^−7^) in nested models. These results were confirmed by the branch model analyses of the other specie sequence set (OSSS). According to these data, the ω of *MnSTS* was higher than that of *MnCHS*, which was consistent with the situation for other specie sequences [ω other *STS* = 0.11261, ω other *CHS* = 0.03729, all the LRT *P* values were much lower than 0.05, except the MnCHS model in OSSS (*P* value = 0.142)]. Because Branch models make the unrealistic assumption of among-site homogeneity [30], we used the Clade and Branch site models in our analyses. The Branch site model detected a very small proportion of p2a (purifying-to-positive selection) and p2b (neutral-to-positive selection) sites in *MnCHS* (MSS: p2a = 0.04242, p2b = 0.00262; OSSS: p2a = 0.05719, p2b = 0.00151) and *MnSTS* (MSS: p2a = 0.12927, p2b = 0.00787; OSSS: p2a = 0.12654, p2b = 0.00331). Notably, the foreground ω of the *MnCHS* and *MnSTS* gene family in two data sets was 1, which was in agreement with the null model with fixed ω2 = 1. Similarly, Clade model C revealed that the majority of sites in the *MnCHS* and *MnSTS* sequences were under purifying selection. In the MSS, about 51.52 % of the p2 sites corresponded to codons that had experienced divergent selection pressures in different partitions. The ω2 of the class sites were 0.12011 for *MnCHS*, 0.28349 for *MnSTS*, and 0.13883 for the background lineages. In the OSSS analysis, this category included 46.76 % of the sites in MnCHS&MnSTS model with ω2 values of 0.17857 (*MnCHS*) and 0.28333 (*MnSTS*). The remaining two alternative hypothesis models also supported these results. The ω2 of p2 sites in MnCHS&oCHS model were 0.15212 for *MnCHS* and 0.06353 for other *CHS*. While in MnSTS&oSTS model, the ω2 of p2 sites were 0.26885 for *MnSTS* and 0.19651 for other *STS*. These results were consistent with the branch model results presented above and statistically significant when the LRT was completed using the M2a_rel null model (all *P* values were much less than 0.05). Moreover, according to the Branch site model, the foreground branch of *MnCHSL2* lacked p2a or p2b sites in both data sets, suggesting selective constraints did not decrease (Additional file 2: Table S3). The ω for the foreground branch of *MnCHSL1* (MSS: ω*MnCHSL1* = 0.1162; OSSS: ω*MnCHSL1* = 0.1302) was higher than that of *MnCHS* lineages (MSS: ω*MnCHS* = 0.0689; OSSS: ω*MnCHS* = 0.0970) according to the Branch model. The proportion of p2 sites (p2 = 1 − p0 − p1) for this foreground branch (MSS: 0.1; OSSS: 0.11) was still higher than that of *MnCHS* lineages (MSS: 0.05; OSSS: 0.06) based on the Branch site model. Similarly, Branch model data indicated the ω for *MnCHSL3* was much higher than that of *MnSTS* genes, and *MnCHSL3* had a higher ω (MSS: 2.885; OSSS: 1.9191) in the Branch site model. The LRT values calculated with this model were not significantly higher than those of the null model, which means positive selection did not occur in this branch. These results indicate that the selective constraints of *MnCHSL1* and *MnCHSL2* were relaxed compared to those of the other genes in the same clades (Fig. 1).

**Table 1:** Parameter Estimates of Codon-Substitution Evolutionary Models for MnCHS and MnSTS family

Model	Proportion of sites			dN/dS ratios			Likelihood	
							lnL	*P*-value
Other specie sequence set								
Branch Model								
MnCHS				ωb = 0.07855 STS + CHSL + oCHS	ωf = 0.09698 MnCHS		−22140.77149	0.1418015 (MnCHS V.S M0)
MnSTS				ωb = 0.07363 CHS + CHSL + oSTS	ωf = 0.16568 MnSTS		−22122.54872	5.192e-010 (MnSTS V.S M0)
oCHS				ωb = 0.09719 STS + CHSL + MnCHS	ωf = 0.03729 oCHS		−22110.96154	3.886e-015 (oCHS V.S M0)
oSTS				ωb = 0.07264 CHS + CHSL + MnSTS	ωf = 0.11261 oSTS		−22132.52527	1.570e-005 (oSTS V.S M0)
MnCHS&MnSTS				ωb = 0.07079 CHSL + oCHS + oSTS	ωf = 0.09885 MnCHS	ωf = 0.16602 MnSTS	−22119.93911	3.595e-011 (MnCHS&MnSTS V.S M0) 3.652e-003 (MnCHS&MnSTS V.S MnCHS-MnSTS)
MnCHS-MnSTS				ωb = 0.07047 CHSL + oCHS + oSTS	ωf = 0.13084 MnCHS-MnSTS		−22124.16361	2.721e-009 (MnCHS-MnSTS V.S M0)
M0				ω0 = 0.08027			−22141.85065	
Clade Model C								
MnCHS&MnSTS	p0 = 0.51866	p1 = 0.01377	p2 = 0.46757	ω0b = 0.02173 CHSL + oCHS + oSTS ω0f1 = 0.02173 MnCHS ω0f2 = 0.02173 MnSTS	ω1b = 1.00000 CHSL + oCHS + oSTS ω1f1 = 1.00000 MnCHS ω1f2 = 1.00000 MnSTS	ω2b = 0.13114 CHSL + oCHS + oSTS ω2f1 = 0.17857 MnCHS ω2f2 = 0.28333 MnSTS	−21704.83735	1.321e-006 (MnCHS&MnSTS V.S M2a_rel)
MnCHS&oCHS	p0 = 0.46637	p1 = 0.01890	p2 = 0.51473	ω0b = 0.02016 CHSL + STS ω0f1 = 0.02016 MnCHS ω0f2 = 0.02016 oCHS	ω1b = 1.00000 CHSL + STS ω1f1 = 1.00000 MnCHS ω1f2 = 1.00000 oCHS	ω2b = 0.16234 CHSL + STS ω2f1 = 0.15212 MnCHS ω2f2 = 0.06353 oCHS	−21695.89064	1.719E-10
MnSTS&oSTS	p0 = 0.47783	p1 = 0.01786	p2 = 0.50430	ω0b = 0.01889 CHSL + CHS ω0f1 = 0.01889 MnSTS ω0f2 = 0.01889 oSTS	ω1b = 1.00000 CHSL + CHS ω1f1 = 1.00000 MnSTS ω1f2 = 1.00000 oSTS	ω2b = 0.11003 CHSL + CHS ω2f1 = 0.26885 MnSTS ω2f2 = 0.19651 oSTS	−21693.48253	1.547E-11
M2a_rel							−21718.37459	
Branch Site Model								
MnCHS	p0 = 0.91716	p1 = 0.02414	p2a = 0.05719 p2b = 0.00151	ω0b = 0.07409 STS + CHSL + oCHS ω0f = 0.05519 MnCHS	ω1b = 1.00000 STS + CHSL + oCHS ω1f = 1.00000 MnCHS	ω2ab = 0.07409 STS + CHSL + oCHS ω2bb,2af,2bf = 1 STS + CHSL + oCHS + MnCHS	−22057.84405	1
MnCHS(NULL)								
MnSTS	p0 = 0.84795	p1 = 0.02220	p2a = 0.12654 p2b = 0.00331	ω0b = 0.07147 CHS + CHSL + oSTS ω0f = 0.07147 MnSTS	ω1b = 1.00000 CHS + CHSL + oSTS ω1f = 1.00000 MnSTS	ω2ab = 0.07147 CHS + CHSL + oSTS ω2bb,2af,2bf = 1 CHS + CHSL + oSTS + MnSTS	−22040.53516	1
MnSTS(NULL)							−22040.53516	
Mulberry sequence set								
Branch Model								
MnCHS				ωb = 0.1264 background	ωf = 0.0689 MnCHS		−9890.922495	1.721E-05 (MnCHS V.S M0)
MnSTS				ωb = 0.0775 background	ωf = 0.1630 MnSTS		−9888.008106	8.220E-07 (MnSTS V.S M0)
MnCHS&MnSTS				ωb = 0.09237 MnPKS + MnCHSL	ωf = 0.07027 MnCHS	ωf = 0.16132 MnSTS	−9886.659146	2.032e-007 (MnCHS&MnSTS V.S M0) 2.290e-007 (MnCHS&MnSTS V.S MnCHS-MnSTS)
MnCHS-MnSTS				ωb = 0.09240 MnPKS + MnCHSL	ωf = 0.10035 MnCHS-MnSTS		−9900.044736	0.630413825 (MnCHS-MnSTS V.S M0)
M0				ω0 = 0.0982			−9900.160494	
Clade Model C								
MnCHS&MnSTS	p0 = 0.45899	p1 = 0.02581	p2 = 0.51519	ω0b = 0.02006 MnCHSL + MnPKS ω0f1 = 0.02006 MnSTS ω0f2 = 0.02006 MnCHS	ω1b = 1.00000 MnCHSL + MnPKS ω1f1 = 1.00000 MnSTS ω1f2 = 1.00000 MnCHS	ω2b = 0.13883 MnCHSL + MnPKS ω2f1 = 0.28349 MnSTS ω2f2 = 0.12011 MnCHS	−9734.263087	1.063e-005 (MnCHS&MnSTS V.S M2a_rel)
M2a_rel							−9745.715191	
Branch Site Model								
MnCHS	p0 = 0.89947	p1 = 0.05549	p2a = 0.04242 p2b = 0.00262	ω0b = 0.07834 background ω0f = 0.07834 MnCHS	ω1b = 1.00000 background ω1f = 1.00000 MnCHS	ω2ab = 0.07834 background ω2bb,2af,2bf = 1 background MnCHS,MnCHS	−9829.594816	1 (MnCHS V.S MnCHS(NULL))
MnCHS(NULL)							−9829.594816	
MnSTS	p0 = 0.81332	p1 = 0.04954	p2a = 0.12927 p2b = 0.00787	ω0b = 0.06667 background ω0f = 0.06667 MnSTS	ω1b = 1.00000 background ω1f = 1.00000 MnSTS	ω2ab = 0.06667 background ω2bb,2af,2bf = 1 background MnSTS,MnSTS	−9813.87732	1 (MnSTS V.S MnSTS(NULL))
MnSTS(NULL)							−9813.87732	
MnCHS-A	p0 = 0.84861	p1 = 0.06802	p2a = 0.07719 p2b = 0.00619	ω0b = 0.05978 MnCHSL + MnSTS ω0f = 0.05978 MnCHS-A	ω1b = 1.00000 MnCHSL + MnSTS ω1f = 1.00000 MnCHS-A	ω2ab = 0.05978 MnCHSL + MnSTS ω2bb = 1, MnCHSL + MnSTS ω2af,2bf = 999.00000 MnCHS-A,MnCHS-A	−8214.284472	3.626E-06 (MnCHS-A V.S MnCHS-A(NULL))
MnCHS-A(NULL)	p0 = 0.83646	p1 = 0.06502	p2a = 0.09141 p2b = 0.00711	ω0b = 0.05774 MnCHSL + MnSTS ω0f = 0.05774 MnCHS-A	ω1b = 1.00000 MnCHSL + MnSTS ω1f = 1.00000 MnCHS-A	ω2ab = 0.05774 MnCHSL + MnSTS ω2bb,2af,2bf = 1 MnCHSL + MnSTS, MnCHS-A,MnCHS-A	−8225.011056	
MnSTS-A	p0 = 0.83361	p1 = 0.06959	p2a = 0.08934 p2b = 0.00746	ω0b = 0.05962 MnCHSL + MnCHS ω0f = 0.05962 MnSTS-A	ω1b = 1.00000 MnCHSL + MnCHS ω1f = 1.00000 MnSTS-A	ω2ab = 0.05962 MnCHSL + MnCHS ω2bb = 1, MnCHSL + MnCHS ω2af,2bf = 210.99468 MnSTS-A,MnSTS-A	−8219.791085	3.684E-02 (MnSTS-A V.S MnSTS-A(NULL))
MnSTS-A(NULL)	p0 = 0.81036	p1 = 0.06741	p2a = 0.11285 p2b = 0.00939	ω0b = 0.05731 MnCHSL + MnCHS ω0f = 0.05731 MnSTS-A	ω1b = 1.00000 MnCHSL + MnCHS ω1f = 1.00000 MnSTS-A	ω2ab = 0.05731 MnCHSL + MnCHS ω2bb,2af,2bf = 1 MnCHSL + MnCHS, MnSTS-A,MnSTS-A	−8221.968483	

The LTR tests are as follow: Branch Modle, One ratio Model 0 vs.Two ratio Model 2. Clade Modle C, M2a_rel vs. CmC. Branch Site Modle, Model A null (w is fixed as 1) vs. Model A. w = dN/dS. b = background; f = foreground; o = other; p0, p1, p2 = proportion of sites with dN/dS ratios = w0, w1, w2, respectively. lnL = ln of the likelihood; *P*-value = *p*-value of the likelihood ratio test

**Fig. 4:**
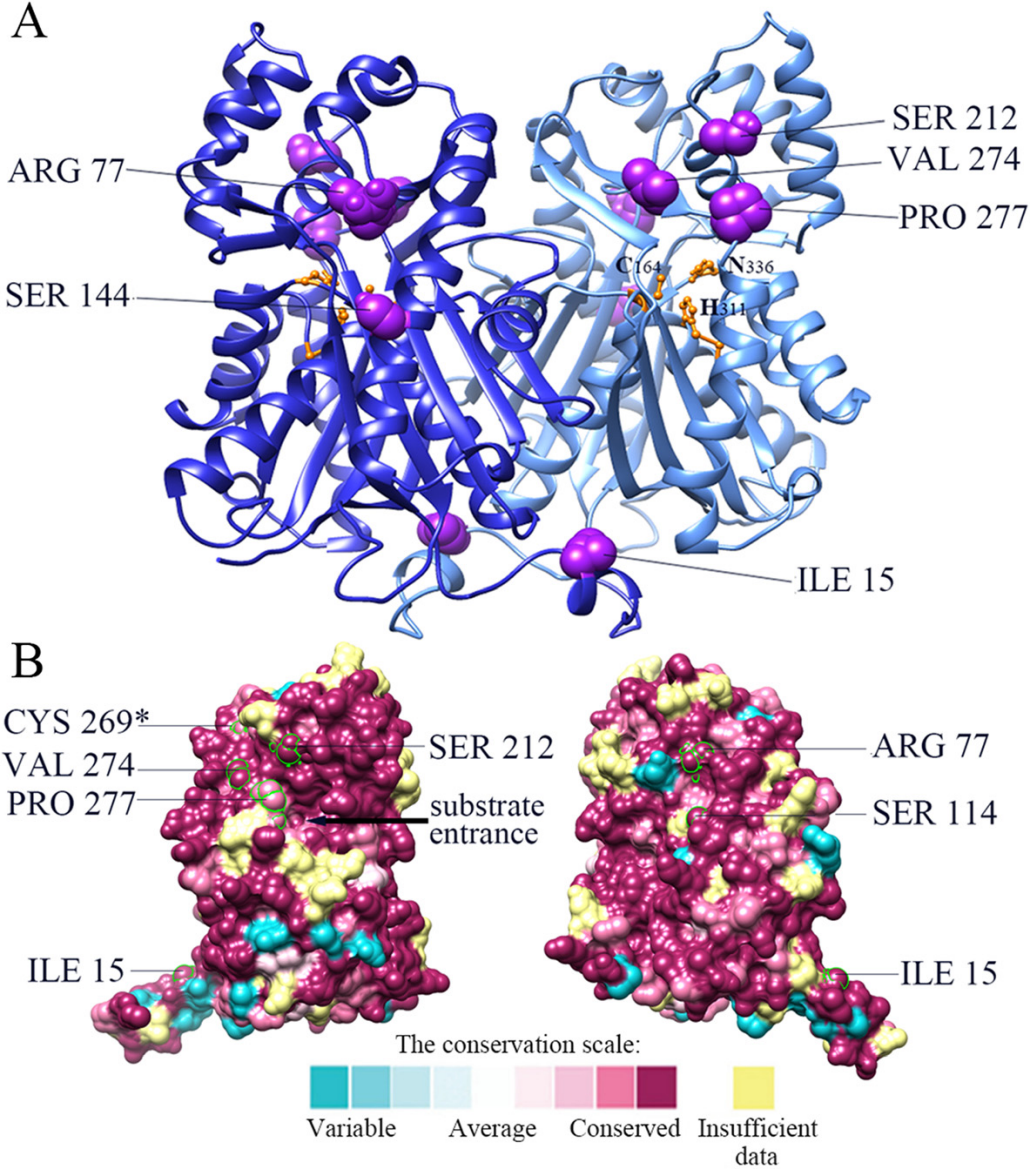
Conserved amino acid sites on a three-dimensional model of a typical STS enzyme. **a** Three dimensional STS dimer structure indicating the conserved amino acid sites. Positive selection and type II divergence sites are shown in purple (numbering is based on MnSTS). Catalytic triads are shown in gold (numbering is based on *Medicago sativa* CHS). **b** Evolutionarily conserved residues in the STS enzyme. All sites are labeled according to their conservation scale. The asterisk indicates the site that experienced positive selection pressure. The remaining six sites underwent positive selection and type II divergence

Because *STS* may have evolved independently from *CHS* several times, positive selection was suspected along the branch ancestral to the *MnSTS* family clade, which corresponds to the foreground branch in our tests (branch b, Fig. 1). Branch site analyses revealed the ancestral branch of the *MnSTS* family experienced strong positive selection (ω approximately 210.99; LRT *P* values <0.05). Bayes empirical Bayes methods were used to calculate the posteriori probability of sites that experienced positive selection. There were seven amino acid sites in branch b with a posteriori probability >0.9 (Additional file 3: Figure S2). ConSurf was then used to calculate the evolutionary conservation of amino acid positions in MnSTS enzymes [31]. All seven positive selection sites were highly conserved, and the majority of these sites existed on the external surface of the MnSTS dimer (Fig. 4b). The positive selection sites Ser-212 and Pro-277 (numbering is based on MnSTS) are associated with CoA-binding and polyketide synthase functional diversity, respectively. This suggests they likely have major roles in MnSTS evolution [8].

### Functional divergence of the mulberry type III PKS

Functional divergence of genes is related to the site-specific change in the corresponding protein sequence during evolution [32]. Therefore, functional divergence among the clusters of mulberry type III PKS was inferred by posterior analysis using the DIVERGE program [33]. Both θ_1_ of type I and θ_2_ of type II functional divergence for the MnSTS subfamily were almost 0 (Additional file 2: Table S4). The results indicated that the coefficients of type I functional divergence (θ_1_) between the MnCHS and MnSTS families were statistically significant (*P* <0.01), with a θ_1_ value of 0.493. Type II functional divergence (θ_2_) between the MnCHS and MnSTS families was also evident with a θ_2_ value of 0.176 (*P* <0.01). A total of seven potential type I sites were detected (posterior probability >0.75). These sites were well conserved in MnCHS clusters, but highly variable in MnSTS clusters (Additional file 3: Figure S2). Of the 31 potential type II sites (posterior probability >4), six were also under positive selection (Fig. 4a and b).

### Functional characterization of selected type III polyketide synthases

**Fig. 5:**
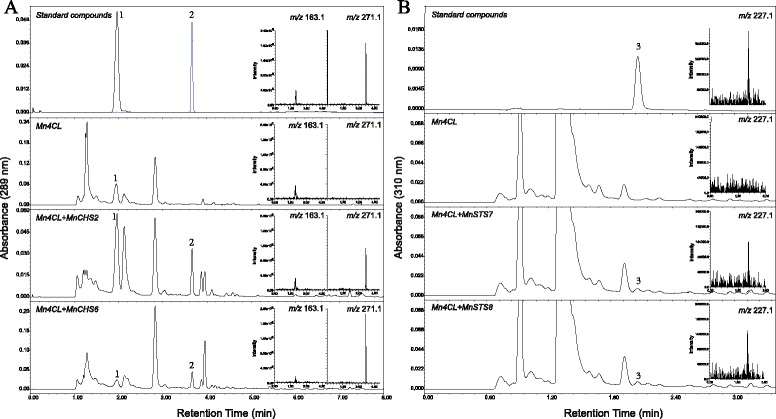
*In vivo* characterization of CHS and STS by coexpression with 4-coumaroyl-CoA ligase in *Escherichia coli.* **a** *In vivo* assay of CHS. 4-Coumaric acid (1) and naringenin (2) were used as standard compounds. *Mn4CL*: *E. coli*-expressed Mn4CL. *Mn4CL + MnCHS2*: *E. coli*-coexpressed Mn4CL and MnCHS2. *Mn4CL + MnCHS6*: *E. coli*-coexpressed Mn4CL and MnCHS6. Selected ion chromatograms generated during liquid chromatography-electrospray ionization mass spectrometry analyses of the compounds are provided in the small panels on the right: 4-coumaric acid, *m*/*z* = 163.1; naringenin, *m*/*z* = 271.1. **b** *In vivo* assay of STS. Resveratrol (3) was used as the standard compound. *Mn4CL*: *E. coli*-expressed Mn4CL. *Mn4CL + MnSTS7*: *E. coli*-coexpressed Mn4CL and MnSTS7. *Mn4CL + MnSTS8*: *E. coli*-coexpressed Mn4CL and MnSTS8. Selected ion chromatograms of the compound are provided in the small panels on the right: resveratrol, *m*/*z* = 227.1

The activities of selected CHS and STS enzymes were assayed *in vivo* by the coexpression with 4-coumaroyl-CoA ligase (4CL) in *Escherichia coli.* 4CL activates 4-coumaric acid to produce 4-coumaroyl-CoA (Additional file 1: Figure S1). When adding phenolic acid starter units to the medium containing transformed *E. coli*, phenylpropionic acids can be efficiently converted to flavonoids or stilbene compounds and secreted into the medium [34, 35]. Four genes, *MnCHS2, MnCHS6*, *MnSTS7*, and *MnSTS8*, were selected for functional analyses, which represented MnCHS-A, MnCHS-B, MnSTS-A, and MnSTS-B groups, respectively (Additional file 5: Figure S4). The addition of *p*-coumaric acid to the growth medium containing *E. coli* cells harboring *Mn4CL* and *MnCHS* resulted in the production of naringenin. Analyses of the medium using LC-MS revealed that the mass of a parent ion (M-H^+^) matched an authentic naringenin standard (*m/z* 271.1) (Fig. 5). Similarly, the expected product, resveratrol (*m/z* 227.1), was also detected after *p*-coumaric acid was added to the growth medium containing *E. coli* cells harboring *Mn4CL* and *MnSTS*. Naringenin and resveratrol peaks were not observed for the medium in which *E. coli* cells carrying only *Mn4CL* were grown (control) (Fig. 5). The MnPKS1 and MnPKS2 enzymatic activities were examined using the same method. *p*-Coumaric acid was not converted to the corresponding naringenin or resveratrol by either MnPKS1 or MnPKS2 (Additional file 6: Figure S5).

In an effort to know the subcellular localization of these genes, their proteins fused with EGFP were transiently expressed in tobacco leaf epidermal cells. As shown in Additional file 7: Figure S6, the fluorescent signal of EGFP alone was detected in the nucleus and cytoplasm. In the cells transformed with MnPKS1-EGFP, MnPKS2-EGFP, MnCHS2-EGFP, MnCHS6-EGFP, MnSTS7-EGFP and MnSTS8-EGFP, fluorescence signals were only detected in the cytoplasm, suggesting that these six proteins were localized to the cytoplasm.

### Expression of mulberry type III *PKS* genes in different tissues

**Fig. 6:**
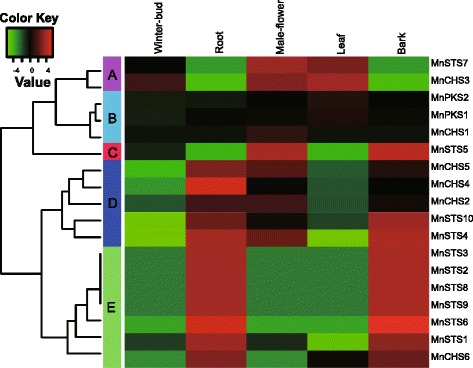
Heat maps of hierarchical clustering of mulberry type III polyketide synthase superfamily genes. Data were adjusted by log transformation and the mean center method. Hierarchical clustering with average linkages was used to calculate K-medians with five clusters

All expressed mulberry type III *PKS* genes were grouped into five clusters using K-medians clustering (Fig. 6). The genes in clusters A, C, D, and E were more highly expressed in five tissues than the genes of cluster B, which were expressed more in leaves or male flowers. *MnCHS3* in cluster A was expressed in leaves, male flowers, and winter buds. *MnCHS6* in cluster E was highly expressed in roots and the bark. Mulberry *CHS* genes in cluster D (i.e., *MnCHS2*, *MnCHS4*, and *MnCHS5*) exhibited relatively high expression levels in roots, male flowers, and the bark. The majority of *MnSTS* genes (i.e., 8 of 10) were expressed in roots and the bark. Only two *MnSTS* genes had a distinct expression profile, namely *MnSTS7* in cluster A with abundant expression in male flowers and leaves, and *MnSTS5* in cluster C with high levels of expression in the bark and male flowers.

### Effect of UV-C irradiation on the expression of mulberry type III *PKS* genes and biosynthesis of the corresponding compounds in mulberry leaves

**Fig. 7:**
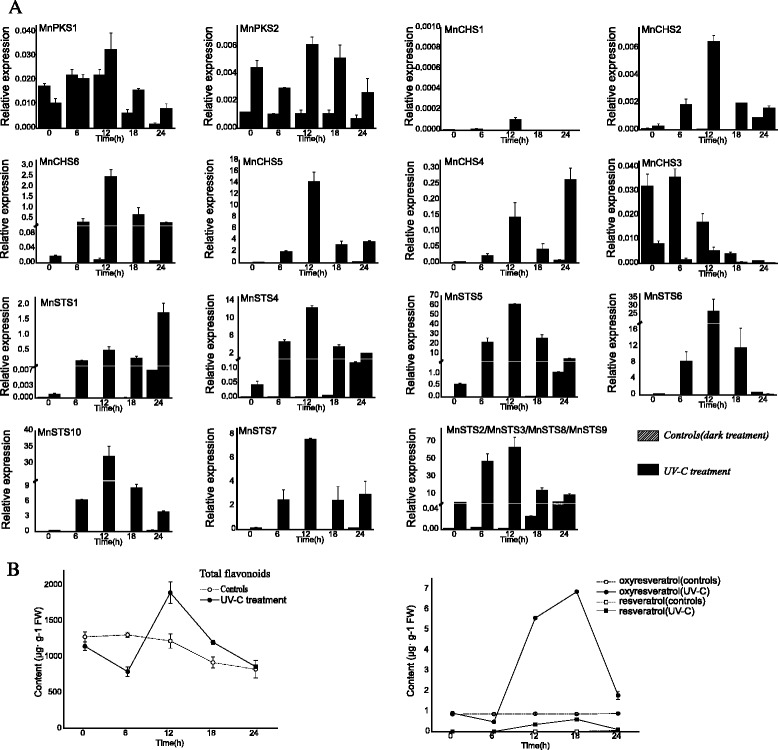
Effect of UV-C irradiation on type III *PKS* gene expression and the biosynthesis of compounds. **a** The *MnSTS2* real-time PCR primers could anneal to the other three *STS* genes (i.e., *MnSTS3*, *MnSTS8*, and *MnSTS9*), although all primers were designed to match the most variable *STS* regions, including the 3′ untranslated regions. Relative gene expression levels were normalized against a mulberry actin gene (*MnACTIN3*). Data are provided as the mean + standard deviation. **b** Variability among compounds following UV-C irradiation. Data are provided as the mean ± standard deviation

The expression of type III *PKS* genes was analyzed in leaves treated with ultraviolet (UV) light. The expression of *MnPKS1* was relatively stable following exposure to UV-C radiation, with a slight decrease 18 h after exposure to the dark (Fig. 7). *MnPKS2* expression increased slightly following UV-C irradiation, and remained stable in the dark. For the *MnCHS* family, UV irradiation resulted in a considerable increase in the expression of three genes in leaves, followed by a decrease after 12 h, especially for *MnCHS5*, whose expression increased about 3.5 × 10^−5^-fold 12 h after UV-C treatment. The expression of *MnCHS4* was highest 24 h after UV-C treatment, and exposure to the dark may have also increased the expression of this gene. In contrast, exposure to UV-C and the dark resulted in a gradual decrease in *MnCHS3* expression. *MnCHS1* was the only gene whose expression was barely detectable even after UV-C exposure. The expression pattern of *MnSTS1* differed from that of the other *MnSTS* genes, with fluctuating profiles that peaked at 24-h after UV-C treatment. The expression levels for the other *MnSTS* genes were highest 12 h after exposure to UV-C radiation. Additionally, treatment in the dark induced the expression of *MnSTS* genes.

Exposure to UV-C radiation resulted in a significant increase (*P* <0.001) in the leaf flavonoid content 12 h after treatment, followed by a decrease in abundance (Fig. 7). The highest total flavonoid content of UV-C treated leaves was 1.6-fold higher than that of control leaves. Additionally, exposure to dark conditions resulted in a decrease in flavonoid abundance. Regarding stilbene content following UV-C irradiation, we measured the accumulation of three representative stilbenes in mulberry: resveratrol (trans-3,4′,5-trihydroxystilbene), oxyresveratrol (trans-2,3′,4,5′-tetrahydroxystilbene, which can be formed by 2′-hydroxylation of resveratrol), and mulberroside A (oxyresveratrol diglycopyranoside, where 4-OH and 3′-OH are replaced by two glucose molecules). Mulberroside A was undetectable in leaves after exposure to UV-C radiation and dark conditions. In contrast, oxyresveratrol content increased 6–18 h after UV-C treatment, peaking at 18 h. Resveratrol was almost undetectable in control samples, even after a slight increase 6–18 h after UV-C irradiation.

## Discussion

### Expansion of the mulberry type III *PKS* gene family

Expansion of multigene families is a common occurrence during evolution, with gene duplications playing an important role [36]. The genomes of terrestrial plants have undergone several gene duplications throughout their evolutionary histories, including tandem, segmental, and whole genome duplications [37, 38]. In this study, six *MnCHS*, ten *MnSTS*, and two *MnPKS* genes were identified in the mulberry genome. *MnPKS1* and *MnPKS2* arose early as ancestors of mulberry type III *PKS* genes. The contention that the number of *MnCHS* genes increased mainly through tandem and segmental duplications is supported by the results of microsynteny analysis. Additionally, tandem duplication is a major reason for the generation of the *MnSTS* family. The transposable elements distributed throughout the *MnSTS* cluster likely had important activities during the formation of tandem arrays. These transposable elements are present in interspersed repetitive elements throughout the genome, and promote crossing over between non-homologous segments during meiosis or recombinational repair [39, 40]. Such unequal recombination may have contributed to the formation of pseudogenes, and a putative gene with undetectable expression, *MnCHSL2*, gained an additional DNA fragment containing a CHS/STS active site (R-[LIVMFYS]-x-[LIVM]-x-[QHG]-x-G-C-[FYNA]-[GAPV]-G-[GAC]-[STAVK]-x-[LIVMF]-[RAL]) during this process.

### The *MnCHS* and *MnSTS* families are under strong purifying selection

The *MnCHS2/MnCHS6* and *MnSTS7*/*MnSTS8* genes were coexpressed with *Mn4CL* in *E. coli*, and the expected products were detected. It is unclear why the mulberry genome contains multiple *MnCHS* and *MnSTS* genes that encode enzymes with redundant catalytic activities. Analyses of evolutionary constraints revealed that no positive selection pressures were associated with *MnSTS* genes, which was consistent with the situation for *MnCHS* genes. Functional divergence within *MnSTS* lineages was also undetected. All studies indicated these two gene families were under strong purifying selection pressure to maintain their functions in the biosynthesis of secondary metabolites. Increasing the number of genes with redundant activities may have been necessary to ensure enough enzymes were produced to synthesize sufficient quantities of phytoalexins in response to abiotic or biotic stresses. According to the seminal theory concerning the fates of duplicate genes, the duplicated copy is shielded from the purifying selection pressure by the ancestral copy [41]. The pseudogenization of the duplicate gene is promoted by decreasing the selective constraint [40, 41]. Compared with those of the *MnCHS* and *MnSTS* families, the selective constraints of *MnCHSL1* and *MnCHSL3* gradually decreased, probably resulting in a lack of *MnCHSL1* and *MnCHSL3* expression.

### Variability in *MnCHS* expression

Phenolic compounds can strongly absorb UV light, which protects plants from DNA damage caused by UV irradiation [13]. In a previous study of grapevine, the transcriptional responses of the *VvSTS* and *VvCHS* genes were diametrically opposed following exposure to UV-C radiation, suggesting that a flow of carbon between these two competing metabolic pathways was tightly regulated at the transcriptional level [42]. However, all *MnSTS* and most *MnCHS* genes were upregulated in mulberry leaves after UV-C treatment. The total flavonoid and stilbene contents exhibited a similar tendency. Only two *MnCHS* genes had distinct expression patterns. *MnCHS1* was almost undetectable in leaves following UV-C treatment. It was expressed exclusively in male flowers, and likely participated in the fruit ripening process [22]. *MnCHS3*, which had the highest expression levels in leaves among *MnCHS* genes, was the only gene whose expression was strongly suppressed after UV-C treatment. The fact that the *MnCHS3* expression levels coincided with total flavonoid contents suggests this gene may be important for flavonoid biosynthesis in mulberry leaves. Additionally, the *MnCHS* genes produced four distinct expression patterns in five tissues, which was in contrast to the *MnSTS* family members, with just two genes exhibiting distinct expression profiles. The evolution of these gene families may allow a fine spatial and temporal regulation of relevant metabolites biosynthesis under both normal and stress conditions, and a greater variability in the expression of *MnCHS* genes may favor plant growth and adaptation to environmental changes.

### Substrate channeling in the stilbene biosynthetic pathway

According to their substrate preferences, STS enzymes are often classified as resveratrol synthase (catalyzes reactions involving 4-coumaroyl-CoA) and pinosylvin synthase (catalyzes reactions involving cinnamoyl-CoA) [14]. In mulberry seedlings of the 7th day to 20th day, the content of resveratrol is higher than that of oxyresveratrol in both leaves and roots, suggesting oxyresveratrol is probably transformed from resveratrol through oxidation [43]. *MnSTS* genes encode enzymes with resveratrol synthase activities, which are involved in producing specific metabolites. Further modifications (e.g., oxidation and O-methylation) of the aromatic ring derived from the phenylpropanoid-CoA substrates occur later [44]. Oxyresveratrol, and not resveratrol, can be detected in fruits, fruit marcs, and leaves of several mulberry species [45]. Additionally, we determined that 2′-hydroxylated resveratrol rapidly accumulated in mulberry leaves following UV-C treatment. An effective transformation pathway converting resveratrol to oxyresveratrol may exist in mulberry plants (Additional file 1: Figure S1). The MnSTS enzymes contain six residues that experienced positive selection and type II functional divergence. All of these sites are highly conserved on the surface, suggesting they may be involved in interacting with other proteins to contribute to channeling resveratrol metabolites in mulberry plants.

## Conclusion

Our results provide new insights into the evolutionary history and the role of mulberry type III *PKS* genes. *MnCHS* genes were expanded mainly through tandem and segmental duplications, while tandem duplications were primarily responsible for the generation of *MnSTS* family. Both *MnCHS* and *MnSTS* genes were under strong purifying selection to maintain their functions during such long-term evolution, suggesting genes with redundant activities may help to increase the abundance of relevant phytoalexins to keep the adaptability of plants in response to abiotic or biotic stresses. In addition, an effective transformation pathway converting resveratrol to oxyresveratrol may exist in mulberry plants. All these results providing the basis for further studies on the biosynthesis of relevant secondary metabolites in mulberry plants and may be useful for improving genomic tools and techniques for genetic manipulation.

## Methods

### Plant materials

The mulberry species, *Morus notabilis* C. K. Schneid., used to clone type III *PKS* genes was grown in Sichuan province, China. For UV-C treatments, mulberry plantlets were grown in a chamber at 25 °C with a 12-h photoperiod until the aerial parts grew to about 20 cm. Seedlings were exposed to UV-C irradiation for 30 min and then maintained in the dark. Control seedlings were kept in the dark without a prior UV-C treatment. Leaves were harvested after 0, 6, 12, 18, and 24 h.

Total RNAs from six tissues (roots, bark, shoots, leaves, male flowers, and winter buds) were extracted using RNAiso Plus (Takara, Otsu, Japan) according to the manufacturer’s instructions. cDNA was synthesized from 1 μg total RNA in a 25 μl reaction using the PrimeScript RT reagent kit (Takara).

### Identification and cloning of mulberry type III *PKS* genes

Previously characterized type III polyketide synthase amino acid sequences were downloaded from UniProt (http://www.uniprot.org/) [46], and the consensus pattern of CHS/STS active site [PS00441] were obtained from PROSITE (http://prosite.expasy.org/) [47]. All sequences were then used as queries in a BLASTP search against the mulberry hypothetical protein database (http://morus.swu.edu.cn/morusdb/) [21]. Mulberry type III *PKS* genes were also identified by TBLASTN analysis against the mulberry genome with an e-value cut-off of 1^e-5^. All hits were analyzed using the Fgenesh++ program (http://www.softberry.com) [21, 48]. The predicted genes were manually corrected through comparisons with other type III *PKS* genes, and were further examined with the online domain analysis program InterProScan (http://www.ebi.ac.uk/interpro/scan.html) [49]. The predicted mulberry type III *PKS* genes were named based on scaffold locations.

Additional file 2: Table S5 lists the primers used in gene cloning. The purified PCR products were cloned into the pMD19-T simple vector (Takara), and the insertions were confirmed by sequencing.

### Phylogenetic analysis of mulberry type III polyketide synthases

The sequences of the enzymes encoded by the type III *PKS* genes were aligned using MUSCLE v. 3.8.31 [50]. The alignment was manually corrected using GeneDoc [51]. Phylogenetic trees were constructed using the neighbor-joining algorithm [52] with the Poisson model and pairwise deletion in MEGA5 [53]. Tree topology was assessed by bootstrap analysis with 1,000 resampling replicates.

### Expansion of the mulberry type III *PKS* superfamily and the timing of duplication events

All scaffolds that contained putative mulberry type III *PKS* genes were analyzed. The microsyntenic regions surrounding genes were detected using MicroSyn software [54]. The mean Ks value was calculated for all pairs of protein-coding genes among all genomic fragments using the method described by Nei and Gojobori [55]. The timing of gene duplication events was estimated using an established procedure, assuming that the average synonymous substitution rate per site per year for plant nuclear genes was about 5 × 10^−9^ [29], i.e., the Ks/(5 × 10^−9^ × 2). Any Ks values greater than 2 were discarded because of the risk of saturation [56]. The MnTEdb was used to search for transposable elements [57].

### Phylogenetic *d*_*N*_*/d*_*S*_ ratio and functional divergence analysis

All full mulberry type III *PKS* coding sequences were aligned using PAL2NAL [58] based on the protein sequence alignment produced by MUSCLE v3.8.31. The phylogenetic trees was generated by Bayesian inference using MrBayes v3.2 [59], the best-fit models for mulberry gene sequence set and multispecies gene sequence set were GTR + G model and SYM + I + G model, respectively. Selection analyses of the type III *PKS* genes were completed using three models from the Codeml program of the PAML v4.3 package [60]. For protein-coding genes, robust evidence for positive selection pressure was provided by a high nonsynonymous substitutions rate *d*_*N*_ relative to the synonymous rate *d*_*S*_ (ω >1). A ω value of 0 or <1 indicated neutral evolution or purifying selection, respectively [61]. Likelihood ratio tests were used to identify the best model for ω changes between two hypotheses. For all LRTs, the null model was a simplified version of the selection model with fewer parameters, while the other model, as the alternative hypothesis, contained *a priori* foreground lineages [62, 63]. The functional divergence of type I and type II genes among the clusters of mulberry type III *PKS* genes was examined using DIVERGE v2.0 [33]. Type-I functional divergence referred to the evolutionary rate shift of site-specific amino acid after gene duplication, whereas Type-II functional divergence referred to the site-specific amino acid physiochemical property shift in the late phase when evolutionary rates were consistent. The *MnCHS*-group A subfamily and *MnPKS* family were excluded because groups with fewer than four sequences could not be analyzed using this method. A θ value >0 indicated altered selective constraints of amino acid sites after gene duplication.

### *In vivo* characterization of a selection of mulberry type III polyketide synthases by coexpression with 4-coumaroyl-CoA ligase in *Escherichia coli*

Six putative type III *PKS* genes, namely *MnPKS1*, *MnPKS2*, *MnCHS2*, *MnCHS6*, *MnSTS7*, and *MnSTS8*, were cloned into a pET28a (+) vector (Novagen) to create pET28a-PKS expression constructs. A pCold-4CL construct was prepared using the pCold vector (Takara) and *Mn4CL*, which encodes an *M. notabilis* 4CL. The primers used to clone *Mn4CL* are listed in Additional file 2: Table S5. Aliquots (5 μl) of overnight cultures of *E. coli* containing pCold-4CL or pCold-4CL and pET28a-PKS were used to inoculate 50 ml Terrific Broth (TB) medium supplemented with ampicillin or kanamycin and ampicillin (100 mg ml^−1^). Cells were grown at 37 °C until the optical density (600 nm) of the cultures reached 0.3. Samples were then cooled on an ice bath for 30 min, and cells were induced with 0.55 mM IPTG at 16 °C. The target recombinant proteins were detected on 12 % SDS–PAGE before processing for further experiments. The cultures were then supplemented with 1 mM 4-coumaric acid prepared in dimethyl sulfoxide. The culture medium was harvested at 24 and 48 h after the addition of the phenolic acid and centrifuged at 13,000 × g for 10 min. The supernatants were divided into two samples, with one (2 ml) acidified with 0.1 N HCl and treated with two volumes of ethyl acetate [35]. The pH of the other sample was adjusted to approximately 9.0 to enable the spontaneous conversion of naringenin chalcone to naringenin *in vitro*, and then treated with an equal volume of ethyl acetate [34, 64]. After evaporating the samples, two extracts were dissolved in 100 μl acetonitrile for analysis by LC-ESI-MS.

### Subcellular localization

A linker (5′-*TGATCCTCCTCCTCCTGATCCTCCTCCTCCTGATCCTCCTCCTCC*-3′) was individually introduced into six type III PKS genes (*MnPKS1*, *MnPKS2*, *MnCHS2*, *MnCHS6*, *MnSTS7*, and *MnSTS8*). The resultant sequences were then inserted into *Kpn*I and *Bam*HI sites of pLGNL-EGFP plasmid to create EGFP-target fusion plasmids. The EGFP-target fusion plasmids and the pLGNL-EGFP control plasmid were introduced into tobacco epidermal cells by *Agrobacterium*-mediated transformation according to a reported method [65]. The signals were detected and photographed under a fluorescent inverted microscope (Olympus IX73, Japan).

### Expression of mulberry type III *PKS* genes

The RNA-Seq data (i.e., SRX504963, SRX504944, SRX504924, SRX504906, and SRX504893) of five mulberry tissuses were downloaded from NCBI. The expression levels of all predicted mulberry type III *PKS* genes were determined according to the fragments per kilo base of transcript per million fragments mapped (FPKM) method [66]. The data were adjusted by log transformation and the mean center method. Hierarchical clustering with average linkages was used to calculate K-medians with five clusters. All data were analyzed and expressed graphically using Heatmap3 [67]. For quantitative reverse transcription PCR (qRT-PCR), the second leaves of mulberry saplings were used. Total RNA extraction and cDNA synthesis were completed as described earlier. Each qRT-PCR was completed using SYBR Premix EX Taq II (Takara) and the StepOnePlus Real-Time PCR System (Applied Biosystems, Foster City, CA, USA) according to the manufacturers’ instructions. Diluted cDNA (2 μl) was used as the template. The mulberry *MnACTIN3* gene served as a control to normalize the target gene expression data. Relative expression was defined as 2^−[Ct (target gene) − Ct (control gene)]^ [68]. Additional file 2: Table S6 lists the gene-specific primers used for qRT-PCR.

### Extraction, identification, and quantification of compounds

Compounds were extracted from the third, fourth, and fifth leaves. Fresh leaf tissue (1 g) in 25 ml 60 % methanol was used in ultrasonic extractions for 40 min. The resulting supernatant was filtered through a membrane with 0.45-μm pores. All samples were prepared in triplicate. To measure the total flavonoid content, 2.5 ml sample was mixed with 1 ml 10 % aluminum chloride and 5 % sodium nitrite. A flavonoid-aluminum complex formed after the addition of 10 ml 1 M sodium hydrate solution. The absorbance at 510 nm was determined after mixing the solution for 5 min [69]. Methanol instead of sample was used as a blank. The total flavonoid content was calculated using a standard curve of rutin [70].

An Acquity UPLC system (Waters, Milford, MA, USA) was used to analyze stilbene content. Separations were performed on an Acquity UPLC BEH C18 column (1.7 μm, 1.0 × 100 mm) at 40 °C. Three standards (i.e., mulberroside A, oxyresveratrol, and resveratrol) were accurately weighed and dissolved in 60 % methanol. Acetonitrile and 0.5 % (v/v) formic acid were used as mobile phases A and B, respectively, with the following elution profile: 0–5 min, 5–30 % A; 5–6 min, 100 % A; 6–7.5 min, 5 % A. Fractions were monitored at 320 nm. Components were identified by comparing the retention times of the eluting peaks with those of commercial standards under the same conditions. Dose-dependent calibration curves of the standards were used to determine the component concentrations.

The products of mulberry type III polyketide synthases were separated using acetonitrile and 0.5 % (v/v) acetic acid as mobile phases A and B, respectively. For the alkaline sample, the gradient elution conditions were as follows: 0–1 min, 30 % A; 1–4 min, 30–80 % A; 4–5 min, 80 % A; 5–7 min, 80–100 % A; 7–8 min, 100–30 % A; 8 min, 30 % A. For the acidic sample, the gradient elution conditions were as follows: 0–1 min, 30 % A; 1–4 min, 30–70 % A; 4 min, 100 % A; 5–6 min, 30 % A. The electrospray ionization mass spectrometer (Acquity QDa Detector) was operated in the negative mode, scanning the mass-to-charge ratio (*m*/*z*) between 100 and 300. Negative ion data for standard compounds were as follows: 4-coumaric acid, *m*/*z* = 163.1; naringenin, *m*/*z* = 271.1; resveratrol, *m*/*z* = 227.1.

### Homology modeling of MnSTS and calculating the evolutionary conservation of amino acid positions

A dimer of the mulberry STS2 enzyme was modeled using MODELER [71]. Structural alignments were prepared using the following known structures: alfalfa (*Medicago. sativa*) CHS enzymes (PDB 1CGZ) [72], peanut STS bound to resveratrol (PDB 1Z1F) [73], and *Freesia hybrida* CHS1 (4WUM) [74]. The generated structure was evaluated using PROCHECK from the Structure Analysis and Verification Server v.4 (http://services.mbi.ucla.edu/SAVES/) [75]. ConSurf (http://consurf.tau.ac.il/) was used to identify the probable evolutionarily conserved residues in the STS enzymes [31]. The analysis was conducted using the Bayesian algorithm with the JTT model.

## Abbreviations

4CL, 4-coumaroyl-CoA ligase; CHS, chalcone synthase; IPTG, isopropyl β-D-1-thiogalactopyranoside; Ks, synonymous silent substitutions per site; LC-ESI-MS, liquid chromatography-electrospray ionization mass spectrometry; LC-MS, liquid chromatography-mass spectrometry; LRT, likelihood ratio test; MSS, mulberry sequence set; MYA, million years ago; OSSS, other specie sequence set; PKS, polyketide synthase; STS, stilbene synthase; TB medium, Terrific Broth medium; UPLC, Ultra Performance Liquid Chromatography; UV-C, ultraviolet-C radiation; ω, the nonsynonymous/synonymous rate ratio.

## Additional files

Additional file 1: Figure S1. General flavonoid and stilbene biosynthetic pathways in mulberry. The enzymes shown in these pathways are as follows: PAL, phenylalanine ammonia-lyase; C4H, cinnamate-4-hydroxylase; 4CL, 4-cumaroyl CoA-lyase; CHS, chalcone synthase; STS, stilbene synthase. (PDF 783 kb) Additional file 2: Table S1. Type III polyketide synthase genes in the *M. notabilis* genome. Genes have been named based on scaffold locations. ORF length: open reading frame length confirmed by clone analysis. *: genes without expression data. Table S2: Estimation of the mean Ks values for the genes flanking mulberry type III *PKS* genes. The mean Ks value was calculated for each homologous pair of protein-coding genes between genomic fragments containing type III *PKS* genes if microsynteny was detected. Table S3: Parameter estimates of codon-substitution evolutionary models for three *MnCHS*-like genes with undetectable expression. The likelihood ratio tests are as follows: Branch model, one ratio model 0 vs two ratio model 2; Clade model C, M2a_rel vs CmC; Branch site model, model A null (ω is fixed as 1) vs model A, ω = *d*_*N*_/*d*_*S*_ *.* b = background; f = foreground; o=other; p0, p1, and p2 = proportion of sites with *d*_*N*_/*d*_*S*_ ratios = ω0, ω1, and ω2, respectively; lnL = ln of the likelihood; P-value = *P* values of the likelihood ratio tests. Table S4: Type I and type II functional divergence between MnSTS and MnCHS families as well as in MnSTS subfamilies. Table S5: Primers used for cloning mulberry type III *PKS* and *4CL* genes. Four genes labelled with asterisk, including *MnSTS2*, *MnSTS3*, *MnSTS8* and *MnSTS9* shared the forward (from initiation codon ‘ATG’) and reverse (end with stop codon ‘TAG’) primers, while other primers matching to variable regions have been used to distinguish them. Table S6: Sequences of primers used for quantitative reverse transcription PCR. (DOCX 36 kb) Additional file 3: Figure S2. Comparison of mulberry type III polyketide synthase amino acid sequences. Conserved sites are shaded. The asterisk indicates the site contains a conserved Thr197 in CHS, but a Gly in PKS1 and PKS2. Purple arrow: positive selection and type II divergence sites; red arrow: positive selection sites; green arrow: type I divergence sites; yellow arrow: type II divergence sites. Red box: catalytic triad; blue box: co-enzyme A binding sites; yellow box: important residues for functional diversity. (PDF 2015 kb) Additional file 4: Figure S3. The phylogenetic tree used to calculate phylogenetic *dN/dS* ratio for multispecies set. (PDF 1102 kb) Additional file 5: Figure S4. Coexpression of *M. notabilis* 4CL and type III PKS in *E. coli*. The results were detected in a SDS-PAGE gel (12%). (A): soluble protein fraction; (B): insoluble protein fraction; Lane 1: pCold-4CL; Lane 2: pCold-4CL+pET28a-MnPKS1; Lane 3: pCold-4CL+pET28a-MnPKS2; Lane 4: pCold-4CL+pET28a-MnCHS2; Lane 5: pCold-4CL+pET28a-MnCHS6; Lane 6: pCold-4CL+pET28a-MnSTS7; Lane 7: pCold-4CL+pET28a-MnSTS8; Lane 8: pCold-4CL control (no IPTG); Lane 9: pCold-4CL+pET28a-MnPKSs control (no IPTG); Lane 10: pCold+pET28a empty plasmids control. Marker: molecular weight standard. (PDF 1187 kb) Additional file 6: Figure S5.
*In vivo* characterization of MnPKS1 and MnPKS2 by coexpression with 4-coumaroyl-CoA ligase in *Escherichia coli. Mn4CL*: *E. coli*-expressed Mn4CL. *Mn4CL+MnPKS1*: *E. coli*-coexpressed Mn4CL and MnPKS1. *Mn4CL+MnPKS2*: *E. coli*-coexpressed Mn4CL and MnPKS2. (A) 4-Coumaric acid (1) and naringenin (2) were used as standard compounds. Selected ion chromatograms generated during liquid chromatography-electrospray ionization mass spectrometry analyses of the compounds are provided in the small panel on the right: 4-coumaric acid, *m*/*z* = 163.1; naringenin, *m*/*z* = 271.1. (B) Resveratrol (3) was used as the standard compound. A selected ion chromatogram of the compound is provided in the small panel on the right: resveratrol, *m*/*z* = 227.1. (PDF 941 kb) Additional file 7: Figure S6. The subcellular localization of mulberry type III polyketide synthases. (A) The control vector pLGNL-EGFP, (B) pLGNL-MnPKS1-EGFP, (C) pLGNL-MnPKS2-EGFP, (D) pLGNL-MnCHS2-EGFP, (E) pLGNL-MnCHS6-EGFP, (G) pLGNL-MnSTS7-EGFP, and (F) pLGNL-MnSTS8-EGFP were transiently expressed in tobacco epidermal cells. Column (a), bright filed; column (b), EGFP fluorescence; and column (c), merged fluorescence. (PDF 3312 kb)
