# Supplementary figures and images for: Evolutionary and functional analysis of mulberry type III polyketide synthases
Source: BMC Genomics. 2016 Aug 4;17:540. doi: 10.1186/s12864-016-2843-7 (PMC4973071; doi:10.1186/s12864-016-2843-7)

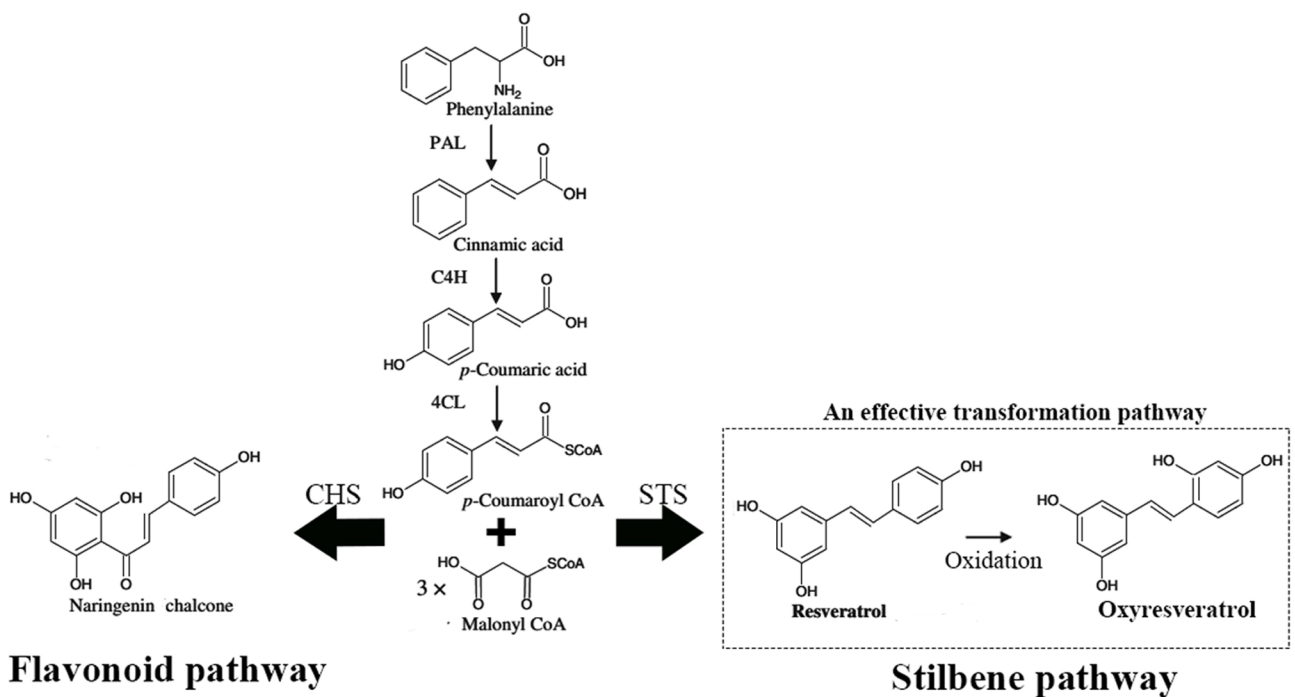

Supplement: Additional file 1: Figure S1. — General flavonoid and stilbene biosynthetic pathways in mulberry. The enzymes shown in these pathways are as follows: PAL, phenylalanine ammonia-lyase; C4H, cinnamate-4-hydroxylase; 4CL, 4-cumaroyl CoA-lyase; CHS, chalcone synthase; STS, stilbene synthase. (PDF 783 kb) [file 12864_2016_2843_MOESM1_ESM.pdf]

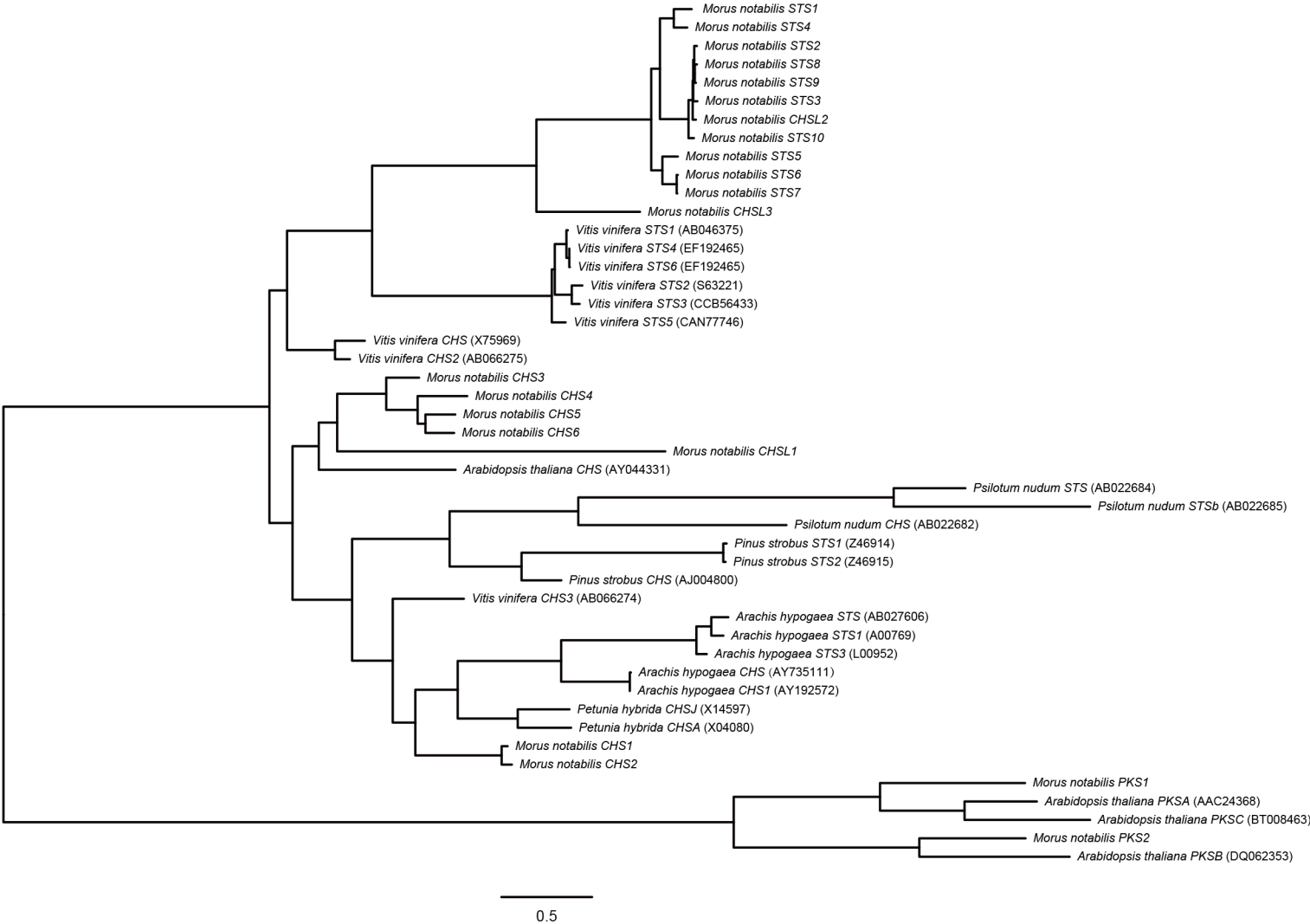

Supplement: Additional file 4: Figure S3. — The phylogenetic tree used to calculate phylogenetic dN/dS ratio for multispecies set. (PDF 1102 kb) [file 12864_2016_2843_MOESM4_ESM.pdf]

(A)

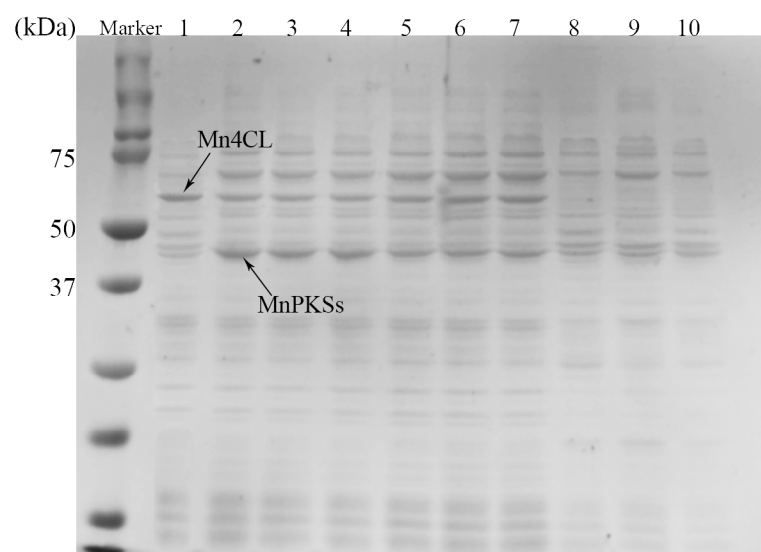

(B)

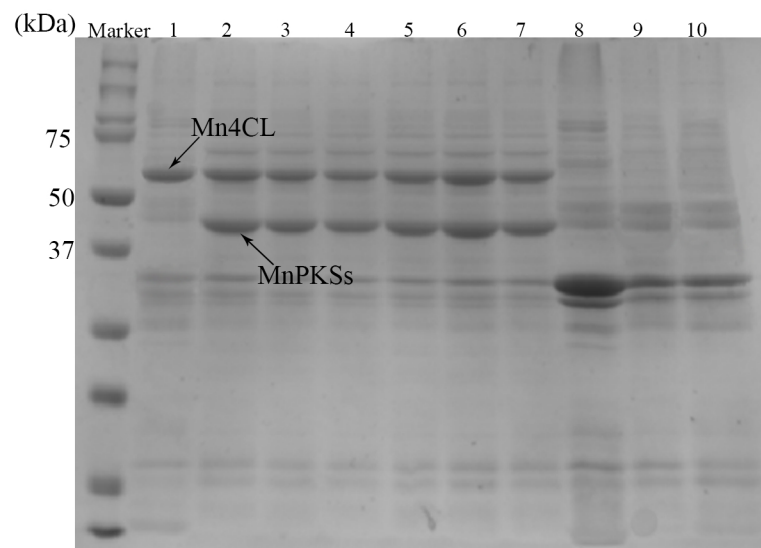

Supplement: Additional file 5: Figure S4. — Coexpression of M. notabilis 4CL and type III PKS in E. coli. The results were detected in a SDS-PAGE gel (12%). (A): soluble protein fraction; (B): insoluble protein fraction; Lane 1: pCold-4CL; Lane 2: pCold-4CL+pET28a-MnPKS1; Lane 3: pCold-4CL+pET28a-MnPKS2; Lane 4: pCold-4CL+pET28a-MnCHS2; Lane 5: pCold-4CL+pET28a-MnCHS6; Lane 6: pCold-4CL+pET28a-MnSTS7; Lane 7: pCold-4CL+pET28a-MnSTS8; Lane 8: pCold-4CL control (no IPTG); Lane 9: pCold-4CL+pET28a-MnPKSs control (no IPTG); Lane 10: pCold+pET28a empty plasmids control. Marker: molecular weight standard. (PDF 1187 kb) [file 12864_2016_2843_MOESM5_ESM.pdf]

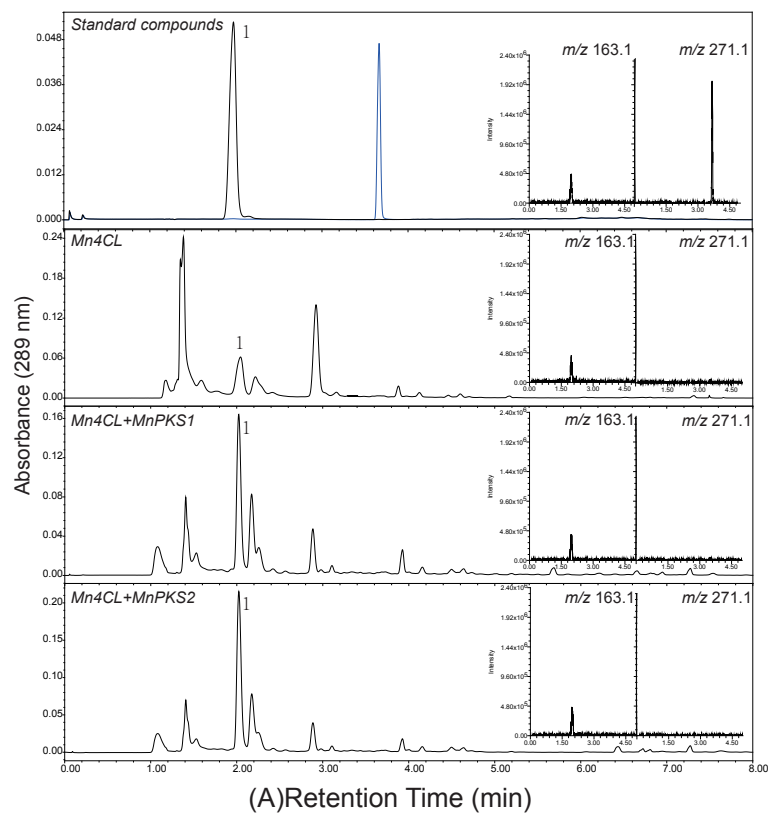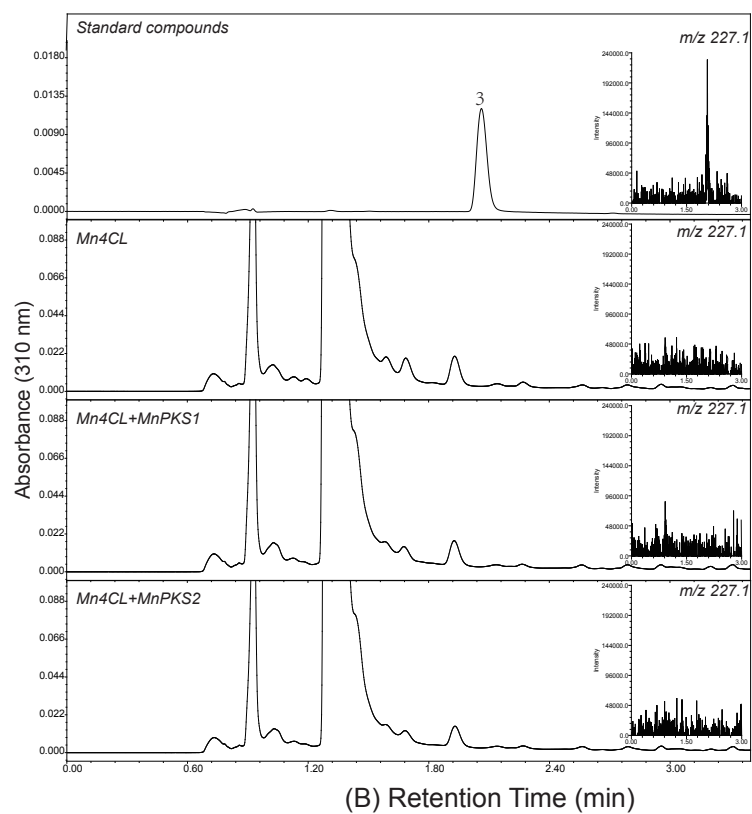

Supplement: Additional file 6: Figure S5. — In vivo characterization of MnPKS1 and MnPKS2 by coexpression with 4-coumaroyl-CoA ligase in Escherichia coli. Mn4CL: E. coli-expressed Mn4CL. Mn4CL+MnPKS1: E. coli-coexpressed Mn4CL and MnPKS1. Mn4CL+MnPKS2: E. coli-coexpressed Mn4CL and MnPKS2. (A) 4-Coumaric acid (1) and naringenin (2) were used as standard compounds. Selected ion chromatograms generated during liquid chromatography-electrospray ionization mass spectrometry analyses of the compounds are provided in the small panel on the right: 4-coumaric acid, m/z = 163.1; naringenin, m/z = 271.1. (B) Resveratrol (3) was used as the standard compound. A selected ion chromatogram of the compound is provided in the small panel on the right: resveratrol, m/z = 227.1. (PDF 941 kb) [file 12864_2016_2843_MOESM6_ESM.pdf]

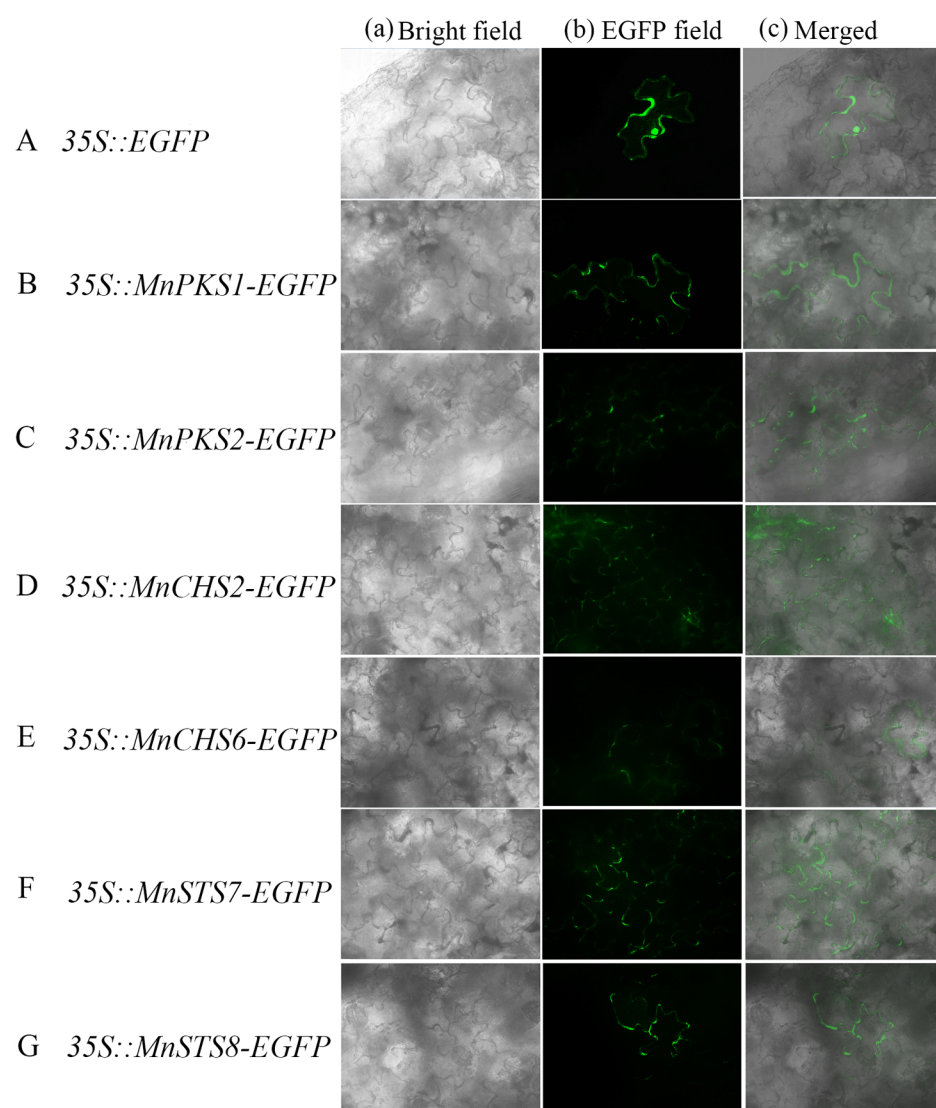

Supplement: Additional file 7: Figure S6. — The subcellular localization of mulberry type III polyketide synthases. (A) The control vector pLGNL-EGFP, (B) pLGNL-MnPKS1-EGFP, (C) pLGNL-MnPKS2-EGFP, (D) pLGNL-MnCHS2-EGFP, (E) pLGNL-MnCHS6-EGFP, (G) pLGNL-MnSTS7-EGFP, and (F) pLGNL-MnSTS8-EGFP were transiently expressed in tobacco epidermal cells. Column (a), bright filed; column (b), EGFP fluorescence; and column (c), merged fluorescence. (PDF 3312 kb) [file 12864_2016_2843_MOESM7_ESM.pdf]
