# Supplementary material for: Evolutionary and functional analysis of mulberry type III polyketide synthases
Source: BMC Genomics. 2016 Aug 4;17:540. doi: 10.1186/s12864-016-2843-7 (PMC4973071; doi:10.1186/s12864-016-2843-7)
Supplement: Additional file 2: Table S1. — Type III polyketide synthase genes in the M. notabilis genome. Genes have been named based on scaffold locations. ORF length: open reading frame length confirmed by clone analysis. *: genes without expression data. Table S2: Estimation of the mean Ks values for the genes flanking mulberry type III PKS genes. The mean Ks value was calculated for each homologous pair of protein-coding genes between genomic fragments containing type III PKS genes if microsynteny was detected. Table S3: Parameter estimates of codon-substitution evolutionary models for three MnCHS-like genes with undetectable expression. The likelihood ratio tests are as follows: Branch model, one ratio model 0 vs two ratio model 2; Clade model C, M2a_rel vs CmC; Branch site model, model A null (ω is fixed as 1) vs model A, ω = d N/d S . b = background; f = foreground; o=other; p0, p1, and p2 = proportion of sites with d N/d S ratios = ω0, ω1, and ω2, respectively; lnL = ln of the likelihood; P-value = P values of the likelihood ratio tests. Table S4: Type I and type II functional divergence between MnSTS and MnCHS families as well as in MnSTS subfamilies. Table S5: Primers used for cloning mulberry type III PKS and 4CL genes. Four genes labelled with asterisk, including MnSTS2, MnSTS3, MnSTS8 and MnSTS9 shared the forward (from initiation codon ‘ATG’) and reverse (end with stop codon ‘TAG’) primers, while other primers matching to variable regions have been used to distinguish them. Table S6: Sequences of primers used for quantitative reverse transcription PCR. (DOCX 36 kb) [file 12864_2016_2843_MOESM2_ESM.docx]

| **Table S1. Identification of the predicted type III polyketide Synthases from mulberry** | | | | | |
| --- | --- | --- | --- | --- | --- |
| Proposed nomenclature | location | ORF length | Exon number | Accession no. | Deduced amino acid number |
| MnCHS1 | scaffold508(+)545726-547456 | 1167 | 2 | KU596023 | 389 |
| MnCHS2 | scaffold508(+)501592-503313 | 1167 | 2 | KU596024 | 389 |
| MnCHS3 | scaffold362(-)57995-59762 | 1173 | 2 | KU596025 | 391 |
| MnCHS4 | scaffold362(-)73939-75445 | 1182 | 2 | KU596026 | 394 |
| MnCHS5 | scaffold398(+)15122-16492 | 1173 | 2 | KU596027 | 391 |
| MnCHS6 | scaffold577(+)205038-206429 | 1173 | 2 | KU596028 | 391 |
| MnSTS1 | scaffold333(-)10176-11961 | 1197 | 2 | KU596031 | 399 |
| MnSTS2 | scaffold643(-)402-2027 | 1197 | 2 | KU596032 | 399 |
| MnSTS3 | scaffold643(-)12794-14347 | 1197 | 2 | KU596033 | 399 |
| MnSTS4 | scaffold643(-)89722-91317 | 1197 | 2 | KU596034 | 399 |
| MnSTS5 | scaffold643(-)104936-106759 | 1197 | 2 | KU596035 | 399 |
| MnSTS6 | scaffold701(-)221658-223426 | 1197 | 2 | KU596036 | 399 |
| MnSTS7 | scaffold701(-)236243-238011 | 1197 | 2 | KU596037 | 399 |
| MnSTS8 | scaffold701(-)243630-245241 | 1197 | 2 | KU596038 | 399 |
| MnSTS9 | scaffold701(-)259333-260893 | 1197 | 2 | KU596039 | 399 |
| MnSTS10 | scaffold966(+)149893-151490 | 1197 | 2 | KU596040 | 399 |
| MnPKS1 | scaffold45(+)1231277-1232851 | 1155 | 2 | KU596029 | 385 |
| MnPKS2 | scaffold1897(-)55904-57179 | 1179 | 2 | KU596030 | 393 |
| MnCHSL1 | scaffold224(-)244985-246722 | 1179* | 2 |  | 393 |
| MnCHSL2 | scaffold643(-)44789-46734 | 1575* | 2 |  | 525 |
| MnCHSL3 | scaffold784(+)32219-33822 | 1188* | 2 |  | 396 |
| Genes have been named based on the scaffold location. ORF: The open reading frame; Number: the length of gene that further confirmed by clone analysis; Asterisk: the genes which undetected their expression in this study. | | | | | |
|  |  |  |  |  |  |
|  |  |  |  |  |  |
|  |  |  |  |  |  |

| **Table S2. Estimation of the mean Ks values for the genes flanking mulberry type III *PKS* genes.** | | | |
| --- | --- | --- | --- |
| genomic fragments | | Ks | Estimated duplication time(million years) |
| MnPKS1 (A) | MnCHS6 (B) | 2.24 | - |
| MnCHS2 (D) | MnCHS6 (B) | 1.73 | 173 |
| MnCHS6 (B) | MnCHSL1 (C) | 1.92 | 192 |
| MnPKS1 (A) | MnCHS2 (D) | 1.17 | 117 |
| MnPKS1 (A) | MnCHS3 (E) | 0.93 | 93 |
| MnCHS2 (D) | MnCHS3 (E) | 1.3 | 130 |
| MnCHS2 (D) | MnCHSL1 (C) | 1.56 | 156 |
| MnCHS2 (D) | MnSTS10 (F) | 0.77 | 77 |
| MnSTS3 (G) | MnSTS8 (H) | 0.37 | 37 |
| The mean Ks value was calculated for each homologous pair of protein-coding genes between genomic fragments containing type III *PKS* genes if microsynteny was detected. | | | |
|  |  |  |  |

| **Table S3. Parameter estimates of codon-substitution evolutionary models for three *MnCHS-Like* genes with undetectable expression.** | | | | | | | | |
| --- | --- | --- | --- | --- | --- | --- | --- | --- |
| Model | Proportion of sites | | | dN/dS ratios | | | Likelihood | |
|  |  |  |  |  |  |  | lnL | P-value |
| **Mulberry sequence set** | | | | | | | | |
| **Branch Model** | | | | | | | | |
| MnCHSL1 |  |  |  | ωb=0.0969 background | ωf=0.1162 MnCHSL1 |  | -9899.935 | 0.501718251 (MnCHSL1 V.S M0) |
| MnCHSL3 |  |  |  | ωb=0.08780 background | ωf=1.3867 MnCHSL3 |  | -9891.024 | 0.000019136 (MnCHSL3 V.S M0) |
| MnCHSL2 |  |  |  | ωb=0.0980 background | ωf=0.1721 MnCHSL2 |  | -9899.966 | 0.532614956 (MnCHSL2 V.S M0) |
| M0 |  |  |  | ω0=0.0982 |  |  | -9900.16 |  |
| **Clade Model C** | | | | | | | | |
| MnCHS&MnCHSL1 | p0=0.43113 | p1=0.02893 | p2=0.53995 | ω0b=0.02206 background ω0f1=0.02206 MnCHS ω0f2=0.02206 MnCHSL1 | ω1b=1.00000 background ω1f1=1.00000 MnCHS ω1f2=1.00000 MnCHSL1 | ω2b=0.19605 background ω2f1=0.10928 MnCHS ω2f2=0.18758 MnCHSL1 | -9739.783 | 0.002652905 (MnCHS&MnCHSL1 V.S M2a_rel) |
| MnSTS&MnCHSL2 | p0=0.46644 | p1=0.02466 | p2=0.50890 | ω0b=0.01965 background ω0f1=0.01965 MnSTS ω0f2=0.01965 MnCHSL2 | ω1b=1.00000 background ω1f1=1.00000 MnSTS ω1f2=1.00000 MnCHSL2 | ω2b=0.12877 background ω2f1=0.28769 MnSTS ω2f2=0.37000 MnCHSL2 | -9734.432 | 0.000012581 (MnSTS&MnCHSL2 V.S M2a_ref) |
| MnSTS&MnCHSL3 | p0=0.45478 | p1=0.02362 | p2=0.52160 | ω0b=0.01784 background ω0f1=0.01784 MnSTS ω0f2=0.01784 MnCHSL3 | ω1b=1.00000 background ω1f1=1.00000 MnSTS ω1f2=1.00000 MnCHSL3 | ω2b=0.11490 background ω2f1=0.24849 MnSTS ω2f2=0.59041 MnCHSL3 | -9727.653 | 0.000000014 (MnSTS&MnCHSL3 V.S M2a_ref) |
| M2a_rel |  |  |  |  |  |  | -9745.715 |  |
| **Branch Site Model** | | | | | | | | |
| MnCHSL1 | p0=0.83265 | p1=0.06750 | p2a=0.09236 p2b=0.00749 | ω0b=0.08155 background ω0f=0.08155 MnCHSL1 | ω1b=1.00000 background ω1f=1.00000 MnCHSL1 | ω2ab=0.08155 background ω2bb,2af,2bf=1 background, MnCHSL1,MnCHSL1 | -9835.726 | 1 (MnCHSL1 V.S MnCHSL1(NULL)) |
| MnCHSL1(NULL) |  |  |  |  |  |  | -9835.726 |  |
| MnCHSL2 | p0=0.93005 | p1=0.06995 | p2a=0.00000 p2b=0.00000 | ω0b=0.08583 background ω0f=0.08583 MnCHSL2 | ω1b=1.00000 background ω1f=1.00000 MnCHSL2 | ω2ab=0.08583 background ω2bb,2af,2bf=1 background MnCHSL2,MnCHSL2 | -9840.851 | 1 (MnCHSL2 V.S MnCHSL2(NULL)) |
| MnCHSL2(NULL) |  |  |  |  |  |  | -9840.851 |  |
| MnCHSL3 | p0=0.59808 | p1=0.03997 | p2a=0.33928 p2b=0.02267 | ω0b=0.07662 background ω0f=0.07662 MnCHSL3 | ω1b=1.00000 background ω1f=1.00000 MnCHSL3 | ω2ab=0.07662 background ω2bb=1, background ω2af,2bf=2.88500 MnCHSL3,MnCHSL3 | -9833.257 | 0.517536429 (MnCHSL3 V.S MnCHSL3(NULL)) |
| MnCHSL3(NULL) |  |  |  |  |  |  | -9833.467 |  |
| **Other specie sequence set** | | | | | | | | |
| **Branch Model** | | | | | | | | |
| MnCHSL1 |  |  |  | ωb=0.07896 background | ωf=0.13016 MnCHSL1 |  | -22139.23 | 0.022035244 |
| MnCHSL2 |  |  |  | ωb=0.08017 background | ωf=0.16884 MnCHSL2 |  | -22141.52 | 0.414940652 |
| MnCHSL3 |  |  |  | ωb=0.07684 background | ωf=0.53116 MnCHSL3 |  | -22130.07 | 1.21E-06 |
| M0 |  |  |  | ω0=0.08027 |  |  | -22141.85 |  |
| **Clade Model C** | | | | | | | | |
| MnCHS&MnCHSL1 | p0=0.52767 | p1=0.01438 | p2=0.45795 | ω0b=0.02345 background ω0f1=0.02345 MnCHS ω0f2=0.02345 MnCHSL1 | ω1b=1.00000 background ω1f1=1.00000 MnCHS ω1f2=1.00000 MnCHSL1 | ω2b=0.14531 background ω2f1=0.16953 MnCHS ω2f2=0.15994 MnCHSL1 | -21717.89 | 0.616251574 (MnCHS&MnCHSL1 V.S M2a_rel) |
| MnSTS&MnCHSL2 | p0=0.51574 | p1=0.01453 | p2=0.46973 | ω0b=0.02191 background ω0f1=0.02191 MnSTS ω0f2=0.02191 MnCHSL2 | ω1b=1.00000 background ω1f1=1.00000 MnSTS ω1f2=1.00000 MnCHSL2 | ω2b=0.13489 background ω2f1=0.28367 MnSTS ω2f2=0.15739 MnCHSL2 | -21706.53 | 0.000007148 (MnSTS&MnCHSL2 V.S M2a_ref) |
| MnSTS&MnCHSL3 | p0=0.51307 | p1=0.01368 | p2=0.47325 | ω0b=0.02151 background ω0f1=0.02151 MnSTS ω0f2=0.02151 MnCHSL3 | ω1b=1.00000 background ω1f1=1.00000 MnSTS ω1f2=1.00000 MnCHSL3 | ω2b=0.13115 background ω2f1=0.26201 MnSTS ω2f2=0.36921 MnCHSL3 | -21701.37 | 0.00000004132 (MnSTS&MnCHSL3 V.S M2a_ref) |
| M2a_rel |  |  |  |  |  |  | -21718.37 |  |
| **Branch Site Model** | | | | | | | | |
|  |  |  |  |  |  |  |  |  |
| MnCHSL1 | p0=0.86074 | p1=0.02468 | p2a=0.11139 p2b=0.00319 | ω0b=0.07636 background ω0f=0.07636 MnCHSL1 | ω1b=1.00000 background ω1f=1.00000 MnCHSL1 | ω2ab=0.07636 background ω2bb,2af,2bf=1 background, MnCHSL1,MnCHSL1 | -22062.34 | 1 (MnCHSL1 V.S MnCHSL1(NULL)) |
| MnCHSL1(NULL) |  |  |  |  |  |  | -22062.34 |  |
| MnCHSL2 | p0=0.97291 | p1=0.02709 | p2a=0.00000 p2b=0.00000 | ω0b=0.07745 background ω0f=0.07745 MnCHSL2 | ω1b=1.00000 background ω1f=1.00000 MnCHSL2 | ω2ab=0.07745 background ω2bb,2af,2bf=1 background MnCHSL2,MnCHSL2 | -22069.96 | 1 (MnCHSL2 V.S MnCHSL1(NULL)) |
| MnCHSL2(NULL) |  |  |  |  |  |  | -22069.96 |  |
| MnCHSL3 | p0=0.73210 | p1=0.01921 | p2a=0.24233 p2b=0.00636 | ω0b=0.07417 background ω0f=0.07417 MnCHSL3 | ω1b=1.00000 background ω1f=1.00000 MnCHSL3 | ω2ab=0.07417 background ω2bb=1, background ω2af,2bf=1.91907 MnCHSL3,MnCHSL3 | -22058.06 | 0.385970134 (MnCHSL3 V.S MnCHSL1(NULL)) |
| MnCHSL3(NULL) |  |  |  |  |  |  | -22058.43 |  |
| The LTR tests are as follow: Branch Modle, One ratio Model 0 vs. Two ratio Model 2; Clade Modle C, M2a_rel vs. CmC; Branch Site Modle, Model A null (w is fixed as 1) vs. Model A. w = dN/dS. b = background; f = foreground; o=other; p0, p1, p2= proportion of sites with dN/dS ratios = w0, w1, w2, respectively. lnL = ln of the likelihood; P-value = p-value of the likelihood ratio test. | | | | | | | | |
|  |  |  |  |  |  |  |  |  |
|  |  |  |  |  |  |  |  |  |

| **Table S4. Type I and type II functional divergence between MnSTS and MnCHS families as well as in MnSTS subfamilies.** | | | | | | | | |
| --- | --- | --- | --- | --- | --- | --- | --- | --- |
|  | Type I | | | | Type II | | | |
|  | ThetaML | SE Theta | LRT Theta | LRT-P | Theta-II | Theta SE | z score | z score-test |
| STS-CHS | 0.49304 | 0.122487 | 8.457903 | 0.0036346 | 0.175864 | 0.038902 | 4.520693 | <0.01 |
| STS-STS | 0.049363 | 0.203136 | 0.030578 | 0.8611358 | -0.011416 | 0.021716 | -0.525695 | 0.29955 |

| **Table S5. Primers used for cloning mulberry type III *PKS* and *4CL* genes**. | |
| --- | --- |
| **Genes** | **Primers (forward/ reverse)** |
| *MnPKS1* | 5'-GTAGAACCCCATCAACCCAAA |
|  | 5'-CCAATTCTTCAAAGGATTCAGC |
| *MnPKS2* | 5'-ACGAAACAAAAACATGGGGAGT |
|  | 5'-AGGTTTTCCTCAGACGGTAGTAGTAA |
| *MnCHS1* | 5'-ATGGTGACCGTCGAGGAAGT |
|  | 5'-CTAAATAGCTACACTGTGGAGCACC |
| *MnCHS2* | 5'-ATGGTGACTGTCGAGGAAGTCT |
|  | 5'-CTAAATAGCTACACTGTGGAGCAC |
| *MnCHS3* | 5'-ATGACGCCCTCCGTCCATGAAAT |
|  | 5'-TTAGACAGCGGGAAGGCTGTGCAG |
| *MnCHS4* | 5'-ATGTCGACACCCTCCTCCGT |
|  | 5'-TTAATTATTGATGGGAAGGCTGTGG |
| *MnCHS5* | 5'-ATGGCAACCTCCGTCCAAGAAAT |
|  | 5'-TTAATTAATGGGAAGGCTACGGAGC |
| *MnCHS6* | 5'-ATGGCGACCTCCGTCCACGAAAT |
|  | 5'-TTAATTAATGGGAAGGCTGTGCAGCACAAC |
| *MnSTS1* | 5'-ATGGCACCGAACAACGTG |
|  | 5'-CTATGCAATAATGGGGACACTTTG |
| *MnSTS2** | 5'-ATGGCGCCGAATAACGTGTCCG |
|  | 5'-CTAAGCAACAATAGGGACACTCTGC |
| *MnSTS3** | 5'-ATGGCGCCGAATAACGTGTCCG |
|  | 5'-CTAAGCAACAATAGGGACACTCTGC |
| *MnSTS4* | 5'-ATGGCGCCAAATAACGTGTCCG |
|  | 5'-CTAAGCAACAATAGGGACACTCTGCAGC |
| *MnSTS5* | 5'-AGTGTTCCCTTAAAGCAGCTA |
|  | 5'-TTACGCAATAATAGGGACACTCTGTAAGAC |
| *MnSTS6* | 5'-GTGTTGCAGAGTGTCCCTATTGT |
|  | 5'-CTATGCAACAATGGGGACGCTCT |
| *MnSTS7* | 5'-ATGGCGCCGACTAACGGGTTC |
|  | 5'-TTATGCAACAATGGGGACACTCTG |
| *MnSTS8** | 5'-ATGGCGCCGAATAACGTGTCCG |
|  | 5'-CTAAGCAACAATAGGGACACTCTGC |
| *MnSTS9** | 5'-ATGGCGCCGAATAACGTGTCCG |
|  | 5'-CTAAGCAACAATAGGGACACTCTGC |
| *MnSTS10* | 5'-TCAACCTTTTAAACTCAGTGTTCCCTT |
|  | 5'-TAGGGACACTCTGCAACA |
| *Mn4CL* | 5'-ATGGCCGATTCAGCGGAT |
|  | 5'-TTAGTTAGGAAAGCCAGCGG |
| Four genes labelled with asterisk, including *MnSTS2*, *MnSTS3*, *MnSTS8* and *MnSTS9* shared the forward (from initiation codon ‘ATG’) and reverse (end with stop codon ‘TAG’) primers, while other primers matching to variable regions have been used to distinguish them. | |
|  |  |

| **Table S6. Sequences of primers used for quantitative reverse transcription PCR.** | | |
| --- | --- | --- |
| **Genes** | **Primers (forward/ reverse)** | **Product size (bp)** |
| *MnPKS1* | 5'-ACCCCAAGACATCACCCAC | 133 |
|  | 5'-GCAGCCGAGGAAGTAGAGCA |  |
| *MnPKS2* | 5'-TTGCCTGGAACAGACAAGACC | 115 |
|  | 5'-TCTCACAGAACCCCTCAACACTA |  |
| *MnCHS1* | 5'-ATGGTGACCGTCGAGGAAG | 204 |
|  | 5'-ACTTCCGCATCACCAATAGTG |  |
| *MnCHS2* | 5'-ATCATGGCAATCGGGACG | 89 |
|  | 5'-TGCTCACTGTTAGTAATACGGAAA |  |
| *MnCHS3* | 5'-ATAATTAATGAGCATGACGCCC | 123 |
|  | 5'-TCAGCCTGTGAGACGAAGTTG |  |
| *MnCHS4* | 5'-AGCTAGCATGTCGACACCCTC | 106 |
|  | 5'-GTAGTTGGGCGGGTTTGC |  |
| *MnCHS5* | 5'-CATTAATTAGCATGGCAACCTCC | 157 |
|  | 5'-TGCTCGCTGTTGGTGATTCG |  |
| *MnCHS6* | 5'-GCGATTGACGGGCATTTG | 247 |
|  | 5'-CGTACTCGCTCAACATCTGACG |  |
| *MnSTS1* | 5'-GCAGCTAGTTATGGCACCGAA | 79 |
|  | 5'-GGCTACTTCATGACCACCTCGT |  |
| *MnSTS2/MnSTS3/MnSTS8/MnSTS9* | 5'-CAGCAATAGCAGCCATCAAA | 137 |
|  | 5'-GACGGATCTTTCGAGGCCAA |  |
| *MnSTS4* | 5'-GGGGTCATGGTGTAGCCTC | 234 |
|  | 5'-ATGGGGCATCATAGGAACAC |  |
| *MnSTS5* | 5'-GAACAACGCTGGAGCACG | 204 |
|  | 5'-ACTTGTGAATTTGAAACGAGCC |  |
| *MnSTS6* | 5'-TTGAGGGCGATTGAAGAGA | 171 |
|  | 5'-CTCCAATCCATCACCAGTG |  |
| *MnSTS7* | 5'-TGGAACTCACTATTTTGGGTG | 210 |
|  | 5'-TCCGTCACCGGTAGTGCT |  |
| *MnSTS10* | 5'-TTGTGGAGATGCCGAAGC | 168 |
|  | 5'-TGACGGATGGTTTGAGGCC |  |
