# Supplementary material for: Evolutionary and functional analysis of mulberry type III polyketide synthases
Source: BMC Genomics. 2016 Aug 4;17:540. doi: 10.1186/s12864-016-2843-7 (PMC4973071; doi:10.1186/s12864-016-2843-7)
Supplement: Additional file 3: Figure S2. — Comparison of mulberry type III polyketide synthase amino acid sequences. Conserved sites are shaded. The asterisk indicates the site contains a conserved Thr197 in CHS, but a Gly in PKS1 and PKS2. Purple arrow: positive selection and type II divergence sites; red arrow: positive selection sites; green arrow: type I divergence sites; yellow arrow: type II divergence sites. Red box: catalytic triad; blue box: co-enzyme A binding sites; yellow box: important residues for functional diversity. (PDF 2015 kb) [file 12864_2016_2843_MOESM3_ESM.pdf]

```
MnSTS1 : MAPNNVPVEESQ-SVIRRGG-HEVASILAIGTANPSNFFNQVDYPDYYFRVTNSEDKTELKEKFKRICEKSLIKKRHMRLTEDILKENPSMCTYDAPSLNARMDLKLVEMPKLGESAAIE : 118
MnSTS2 : MAPNNVSVEGSQ-PVIRRGG-PGVASILAIGTANPDNFFNQADYPDYYFRVTNSEDKTELKEKFKRICEKSLIKKRHMRLTEDILKENPSMCSYDAPSLNARMDLKLVEMPKLGESAAIA : 118
MnSTS3 : MAPNNVSVEGSQ-PVIRRGG-PGVASILAIGTANPDNFFNQADYPDYYFRVTNSDDKTELKEKFKRICEKSLIKKRHMRLTEDILKENPSMCSYDAPSINARMDLKLVEMPKLGESAAIA : 118
MnSTS4 : MAPNNVSVEGSQ-TVIQRGG-HGVASILAIGTANPSNFFIQADYPDYYFRVTNSEDKTELKEKFKRICEKSLIKKRHMRLTEDILKENPSMCSYDAPSINERMDLKLVEMPKLGESAAIE : 118
MnSTS5 : MAPNNVSVEQSQ-TVIPRGG-HAVASILAIGTANPDNFFNQADYPDYYFRVTNSEHKTELKEKFKRICEKSLIKKRHMRLTEEILKENPSICTYDAPSINERMDLKIVEMPKLGESAAIA : 118
MnSTS6 : MAPTNGFVEESQ-TVIPRAG-PAVASILAIGTSNPSNYFNQAEYADYYFRVTNSEHMTELKEKFKRICEKSLIKKRHMRLTEDILKANPSICTYDGPSINERMDLKIVEMPKLGESAAIE : 118
MnSTS7 : MAPTNGFVEESQ-TVIPRAG-PAVASILAIGTSNPSNYFNQAEYADYYFRVTNSEHMTELKEKFKRICEKSLIKKRHMRLTEDILKANPSICTYDGPSINERMDLKIVEMPKLGESAAIE : 118
MnSTS8 : MAPNNVSVEGSQ-PVIRRGG-PGVASILAIGTANPDNFFNQADYPDYYFRVTNSEDKTELKEKFKRICEKSLIKKRHMRLTEDILKENPSMCSYDAPSLNARMDLKLVEMPKLGESAAIA : 118
MnSTS9 : MAPNNVSVEGSQ-PVIRRGG-PGVASILAIGTANPDNFFNQADYPDYYFRVTNSEDKTELKEKFKRICEKSLIKKRHMRLTEDILKENPSMCSYDAPSLNARMDLKLVEMPKLGESAAIA : 118
MnSTS10: MAPNNVSVEGSQ-PVIRRGG-PAVASILAIGTANPDNFFNQADYPDYYFRVTNSEDKTELKEKFKRICEKSLIKKRHMRLTEDILKENPSMCTYDAPSLNARMDLKLVEMPKLGESAAIA : 118
MnCHS1 : ----MVTVEEVR-KAQRAEG-P--VTIMAIGTATPPNCIDQSSYPDYYFRITNSEHKTELKEKFKRMCEKSQIKKRYMYLHEEILKENPNICAYMAPSLDARQDMVVVEVPKLGKEAATK : 112
MnCHS2 : ----MVTVEEVC-RAQRAEG-P--ATIMAIGTANPPNCFDQRTYPDFYFRITNSEHKTELKEKFKRMCEKSLIKKRYMYLHEEILKENPNICAYMAPSLDARQDMVVVEVPKLGKEAATK : 112
MnCHS3 : MTP---SVHEIR-KAQRSEG-P--ATVLSIGTATPTNFVSQADYPDYYFRITNSDHMTDLKDKFKRMCEKSMITKRHMYLTEEILKENPKMCEYMAPSLDARQDIVVVEVPKLGKEAAAK : 113
MnCHS4 : -MSTPSSVQEIR-KAQRADG-P--AAVLAIGTANPPNYFFQTDYPDFYFHITNSDHKTELKDKFTRMCEKAMIRKRHMYLTEEILKENPKMCEFMAPSLDARQDIVVVEIPKLGKEAAVK : 115
MnCHS5 : ---MATSVQEIR-KAQRAEG-L--AAVLAIGTANPPNFFMQADYPDFYFRITNSEHKTELKHKFRRMCEKAMIRKRHMYLTEEILNENPKMCEYMAPSLDARQDIVVVEIPKLGKEAAVK : 113
MnCHS6 : ---MATSVHEIR-KAQRAEG-P--AAVLAIGTANPPNFFMQADYPDFYFRITNSEHKTELKDKFRRMCEKATIKKRHMYLTEEILKENPKMCEYMAPSLDARQDIVVVEIPKLGKEAAVK : 113
MnPKS1 : ----MSRVEGSEAPRPRRIPTPGRATILAIGKAFPSQLIPQECLVEGYIRDTNCQD-ASIKEKLERLCKTTTVKTRYTVMCKDILEKYPELAREGTPTINQRLEIANPAVVEMAKEASLA : 115
MnPKS2 : ----MGSLDDAR-LTSPKKATPGKATILALGKAFPHQLVMQEYLVDGYFRDTNCDD-PELKRKLTRLCKTTTVKTRYVVMSEEILKKYPELALEGRSTVKQRLDICNDAVTQMAIEASQA : 114

MnSTS1 : AIKEWGQPKSKITHIIVNSTSGVDMPGADYQLIRSLGLKTSVKRVMLYHQGCFAGGTVLRIAKDLAENNPGARVLVVCSELTIPTFRAPSEDDSASLVGQAIFADGGSAVIIGAN-VPDE : 237
MnSTS2 : AIKEWGQPKSKITHLIVNSTSGVDMPGADYQLIKSLGLERSVKRVMLYHQGCFAGGTVLRIAKDLAENNPGSRVLVVCSELTIPTFRGPSEDDSASLVGQAIFADGASAVIVGAN-VPDE : 237
MnSTS3 : AIKEWGQPKSKITHLIVNSTSGVDMPGADYQLIKSLGLERSVKRVMLYHQGCFAGGTVLRIAKDLAENNPGSRVLVVCSELTIPTFRGPSEDDSASLVGQAIFADGASAVIVGAN-VPDE : 237
MnSTS4 : AIKEWGQPKSKITHIIVNSTSGVDMPGADYQLIRSLGLKTSVKRVMLYHQGCFAGGTVLRIAKDLAENNPGARVLVVCSELTIPTFRGPSEDDSASLVGQAIFADGGSAVIIGAN-VPDE : 237
MnSTS5 : ALKEWGQPKSKITHIIVNSTSGVDMPGADYQLIRSLGLKTSVKRVMLYHQGCFAGGTVLRIAKDLAENNAGARVLVVCSELTIPTFRAPSEDDSASLVGQAIFADGGSAVIVGAN-VPDE : 237
MnSTS6 : ALKEWGQPKSKITHIIVNSTSGVDMPGADYQLIRSLGLKTSVKRVMLYHQGCFAGGTVLRIAKDLAENNPGARVLVVCSELTIPTFRGPSEEDSASLVGQAIFADGGSAVIVGAN-VPDE : 237
MnSTS7 : ALKEWGQPKSKITHIIVNSTSGVDMPGADYQLIRSLGLKTSVKRVMLYHQGCFAGGTVLRIAKDLAENNPGARVLVVCSELTIPTFRGPSEEDSASLVGQAIFADGGSAVIVGAN-VPDE : 237
MnSTS8 : AIKEWGQPKSKITHLIVNSTSGVDMPGADYQLIKSLGLERSVKRVMLYHQGCFAGGTVLRIAKDLAENNPGSRVLVVCSELTIPTFRGPSEDDSASLVGQAIFADGASAVIVGAN-VPDE : 237
MnSTS9 : AIKEWGQPKSKITHLIVNSTSGVDMPGADYQLIKSLGLERSVKRVMLYHQGCFAGGTVLRIAKDLAENNPGSRVLVVCSELTIPTFRGPSEDDSASLVGQAIFADGASAVIVGAN-VPDE : 237
MnSTS10: AIKEWGQPKSKITHLIVNSTSGVDMPGADYQLIKSLGLKPSVKRVMLYHQGCFAGGTVLRIAKDLAENNPGSRVLVVCSELTIPTFRGPSEDDSASLVGQAIFADGASAVIVGAN-VPDE : 237
MnCHS1 : AIKEWGQPKSKITHLVFCTTSGVDMPGADYQLTKLLGLRPSVKRLMMYQQGCFAGGTVLRLAKDLAENNKGARVLVVCSEITAVTFRGPSDTHLDSLVGQALFGDGAAAMIIGADPIPE- : 231
MnCHS2 : AIKEWGQPKSKITHLVFCTTSGVDMPGADYQLTKLLGLRPSVKRLMMYQQGCFAGGTVLRLAKDLAENNKGARVLVVCSEITAVTFRGPSDTHLDSLVGQALFGDGAAAMIIGADPIPE- : 231
MnCHS3 : AIKEWGQPKSKITHLIFCTTSGVDMPGADYQLTKLLGLRPSVKRFMMYQQGCFAGGTVLRLAKDLAENNKGARVLVVCSEITAVTFRGPSHTHLDSLVGQALFGDGAAAVIVGAD--PDT : 231
MnCHS4 : ALKEWGQPKSKITHLIVCTTSGVDMPGADYQITKLLGLRPSVKRFMLYQQGCFAGGAVLRLAKDLAENNKGARVLVVCSEITAVTFRGPSELHLDSMVGQALFGDGAAAVIVGAE--PDL : 233
MnCHS5 : ALKEWGQPKSKITHLIVCTTSGVDMPGADYQLLKLLGLRPSVRRFMMYQQGCFAGGAVLRLAKDMAENNKGARVLVVCSEITAVTFRGPSELHLDSMVGQALFGDGAAAVIVGAE--PDL : 231
MnCHS6 : AIKEWGQPKSKITHLIVCTTSGVDMPGADYQITKLLGLRPSVKRFMMYQQGCFAGGAVLRLAKDLAENNKGARVLVVCSEITAVTFRGPSELHLDSMVGQALFGDGAAAVIVGAE--PDL : 231
MnPKS1 : CIKEWGRPPQDITHIVYVSSSEIRLPGGDLHLASQLGLRTDVGRVMLYFLGCYGGVTGLRVAKDIAENNPGSRVLLTTSETTILGFRPPNMSRPYDLVGAALFGDGAAAVIIGAD--PIT : 233
MnPKS2 : CIKNWGRPVSDITHLVYVSSSEIRLPGGDLYLASGLGLSPETQRVMLYFVGCSGGVAGLRVAKDIAENNPGSRILLATSETTIIGYKPPSADRPYDLVGVALFGDGAGAMIIGSD--PVS : 232
                                                                                                        *

MnSTS1 : GSVERPLFRLISTSEVILPNSENTIGGHLRDCGLTIVLSPEVPKLIGKNILTCLEEAFTPYGIS-DW-NSVFWVPHPGGAAILRAIEEKAELKKEKLKDTWNVWSEYGNMSSATVFFILN : 355
MnSTS2 : GSVERPLFRLVSTSEVILPNSENTVGGHLRDCGLTIVLSPQVPKIIGKNIQTCLEEALGPFGIS-DW-NSVFWAPHPGGAAIIKEIEEKAGLEKEKLKDTWNVWSEYGNMSSATVFFILN : 355
MnSTS3 : GSVERPLFRLVSTSEVILPNSENTVGGHLRDCGLTIVLSPQVPKIIGKNIQTCLEEALGPFGIS-DW-NSVFWAPHPGGAAIIKEIEEKAGLEKEKLKDTWNVWSEYGNMSSATVFFILN : 355
MnSTS4 : GSVERPLFRLISTSEVILPNSENTVGGHLRDCGLTIVLSPEVPKLIGKNIQTCLEEAFSPYGIS-DW-NSVFWAPHPGGAAIIKEIEEKAGLEKEKLKDTWNVWSEYGNMSSATVFFILN : 355
MnSTS5 : GSVERPLFRLVSNSQVILPNSENTVGGHLRDCGLTIVLSPEVPKLIGKNIQTCLEEAFTPFGIS-DW-NSLFWVPHPGGAAILRAIEEKAGLEKEKLKDTWNVWSEYGNMSSATVFFILN : 355
MnSTS6 : GSVERPLFRLVSNSQVILPNSENTVGGHLRDCGLTIVLSPEVPKLIGKNILTCLEEAFTPFGIS-DW-NSLFWVPHPGGAAILRAIEEKAELKKEKLKDTWNVWSEYGNMSSATVFFILN : 355
MnSTS7 : GSVERPLFRLVSNSQVILPNSENTVGGHLRDCGLTIVLSPEVPKLIGKNILTCLEEAFTPFGIS-DW-NSLFWVPHPGGAAILRAIEEKAELKKEKLKDTWNVWSEYGNMSSATVFFILN : 355
MnSTS8 : GSVERPLFRLVSTSEVILPNSENTVGGHLRDCGLTIVLSPQVPKIIGKNIQTCLEEALGPFGIS-DW-NSVFWAPHPGGAAIIKEIEDKAGLEKEKLKDTWNVWSEYGNMSSATVFFILN : 355
MnSTS9 : GSVERPLFRLVSTSEVILPNSENTVGGHLRDCGLTIVLSPQVPKIIGKNIQTCLEEALGPFGIS-DW-NSVFWAPHPGGAAIIKEIEEKAGLEKEKLKDTWNVWSEYGNMSSATVFFILN : 355
MnSTS10: GSVERPLFRLVSTSEVILPNSENTVGGHLRDCGLTIVLSPQVPKIIGKNIQTCLEEALGPLGIN-DW-NSVFWVPHPGGAAIIKEIEEKAGLEKEKLKDTWNVWSEYGNMSSATVFFILN : 355
MnCHS1 : --VEKPILQLVSAAQTILPDSEGAIDGHLREVGLTFHLLKDVPGLISKNIDKSLTEAFKPFGIS-DW-NSIFWIAHPGGPAILDQVEAKCGLKPEKLRATRHVLSEYGNMSSACVVFILD : 347
MnCHS2 : --VEKPIFQLVSAAQTILPDSEGAIDGHLREVGLTFHLLKDVPGLISKNIDKSLTEAFKPFGIS-DW-NSIFWIAHPGGPAILDQVEAKCGLKPEKLRATRHVLSEYGNMSSACVVFILD : 347
MnCHS3 : -SVERPIFELVSAAQTILPDSEGAIDGHLREVGLTFHLLKDVPGLISKNIEKSLVEAFTPIGIS-DW-NSIFWIAHPGGPAILDQVETKLGLKQEKLSATRHVLSEYGNMSSACVLFILD : 348
MnCHS4 : -SIERPIFELVSAAQTILPDSEGAIDGHLREIGLTFHLLKDVPLLISKNIEKSLEEAFTPIGIS-DW-NSIFWIAHPGGPAILDQVEAKVGLHKEKLRATREMLREYGNMSSASVLFILD : 350
MnCHS5 : -AIERPLFELVSAAQTILPDSEGAIDGHLREVGLTFHLLKDVPMLISKNIGKSLEEAFTPLGIS-DW-NSIFWVAHPGGPAILDQVEATVGLKKEKLRATREMLKEYGNMSSASVLFILD : 348
MnCHS6 : -SIERPLFELVSAAQTILPDSEGAIDGHLREVGLTFHLLKDVPALISKNIEKSLVEAFTPIGIS-DW-NSIFWVAHPGGPAILDQVEAKVGLKKEKLTATRQMLSEYGNMSSASVLFILD : 348
MnPKS1 : -DLESPFMELNSAVQQFLPNTHDVIDGKLSEEGINFRLGRDLPQKIEDNIEEFCKKLMAKAGLKFEY-NELFWAVHPGGPAILNKLESCLKLRSEKLVSSRRALMDYGNVSSNTILYVIE : 351
MnPKS2 : -ETEKPLFELHSAMQRFLPGTDKTIDGRLTEEGISFKLARELPQVIEDSVEGFCEKLMGVKGFDKEWYNKMFWAVHPGGPAILNRIEKRLDLDPEKLNASRKALMDYGNASSNTIVYVLE : 351

MnSTS1 : QMRKRSLAEKKSTTGDGLEWGVLLGFGPGLTVETVVLQSVPIIA : 399
MnSTS2 : QMRKRSLEEKKSTTGDGLEWGVLLGFGPGLTVETVVLQSVPIVA : 399
MnSTS3 : QMRKRSLEEKKSTTGDGLEWGVLLGFGPGLTVETVVLQSVPIVA : 399
MnSTS4 : QMRKRSLEEKKSTTGDGLKWGVLLGFGPGLTVETVVLQSVPIVA : 399
MnSTS5 : QMRKRSLEEKKSTTGDGLEWGVLLGFGPGLTVETVVLQSVPIIA : 399
MnSTS6 : QMRKRSLEEKKSTTGDGLEWGVLLGFGPGLTVETVVLQSVPIVA : 399
MnSTS7 : QMRKRSLEEKKSTTGDGLEWGVLLGFGPGLTVETVVLQSVPIVA : 399
MnSTS8 : QMRKRSLEEKKSTTGDGLEWGVLLGFGPGLTVETVVLQSVPIVA : 399
MnSTS9 : QMRKRSLEEKKSTTGDGLEWGVLLGFGPGLTVETVVLQSVPIVA : 399
MnSTS10: QMRKRSLEEKKSTTGDGLEWGVLLGFGPGLTVETVVLQSVPIVA : 399
MnCHS1 : EMRRKGAEDGLKTTGEGLEWGVLFGFGPGLTVETVVLHSVAI-- : 389
MnCHS2 : EMRRKGAEDGLKTTGEGLEWGVLFGFGPGLTVETVVLHSVAI-- : 389
MnCHS3 : EMRKKSVEEGKATTGEGLEWGVLFGFGPGLTVETVVLHSLPAV- : 391
MnCHS4 : EMRKKSAEEGKSTTGEGLDWGVLFGFGPGLTVETVVLHSLPINN : 394
MnCHS5 : EMRKKSAEEAKSTTGEGLDWGVLFGFGPGLTVETVVLRSLPIN- : 391
MnCHS6 : EMRKKSAEEGKSTTGEGLEWGVLFGFGPGLTVETVVLHSLPIN- : 391
MnPKS1 : NMREELKREGSE------EWGFGLAFGPGITFEGILIRSL---- : 385
MnPKS2 : YMLEES--SKMKVDQEENEWGLILAFGPGITFEGILARNLTTTV : 393
```
